# New insights into the giant mustelids (Mammalia, Carnivora, Mustelidae) from Langebaanweg fossil site (West Coast Fossil Park, South Africa, early Pliocene)

**DOI:** 10.7717/peerj.9221

**Published:** 2020-06-01

**Authors:** Alberto Valenciano, Romala Govender

**Affiliations:** 1 Department of Research and Exhibitions, Iziko Museums of South Africa, Cape Town, South Africa; 2 Department of Biological Science, University of Cape Town, Cape Town, South Africa

**Keywords:** Miocene, Pliocene, Neogene, Lutrinae, Guloninae, Carnivora, Africa

## Abstract

Giant mustelids are a paraphyletic group of mustelids found in the Neogene of Eurasia, Africa and North America. Most are known largely from dental remains, with their postcranial skeleton mostly unknown. Here, we describe new craniodental and postcranial remains of the large lutrine *Sivaonyx hendeyi* and the leopard-size gulonine *Plesiogulo* aff. *monspessulanus* from the early Pliocene site Langebaanweg, South Africa. The new material of the endemic *S. hendeyi*, includes upper incisors and premolars, and fragmentary humerus, ulna and a complete astragalus. Its postcrania shares more traits with the living *Aonyx capensis* than the late Miocene *Sivaonyx beyi* from Chad. *Sivaonyx hendeyi* could therefore be tentatively interpreted as a relatively more aquatic taxon than the Chadian species, comparable to *A. capensis*. The new specimens of *Plesiogulo* comprise two edentulous maxillae, including one of a juvenile individual with incomplete decidual dentition, and a fragmentary forelimb of an adult individual. The new dental measurements point to this form being amongst the largest specimens of the genus. Both P3-4 differs from the very large species *Plesiogulo botori* from late Miocene of Kenya and Ethiopia. This confirms the existence of two distinct large species of *Plesiogulo* in Africa during the Mio/Pliocene, *P. botori* in the Late Miocene of Eastern Africa (6.1–5.5 Ma) and *Plesiogulo* aff. *monspessulanus* at the beginning of the Pliocene in southern Africa (5.2 Ma). Lastly, we report for the first time the presence of both *Sivaonyx* and *Plesiogulo* in MPPM and LQSM at Langebaanweg, suggesting that the differences observed from the locality may be produced by sedimentation or sampling biases instead of temporal replacement within the carnivoran guild.

## Introduction

**Figure 1:**
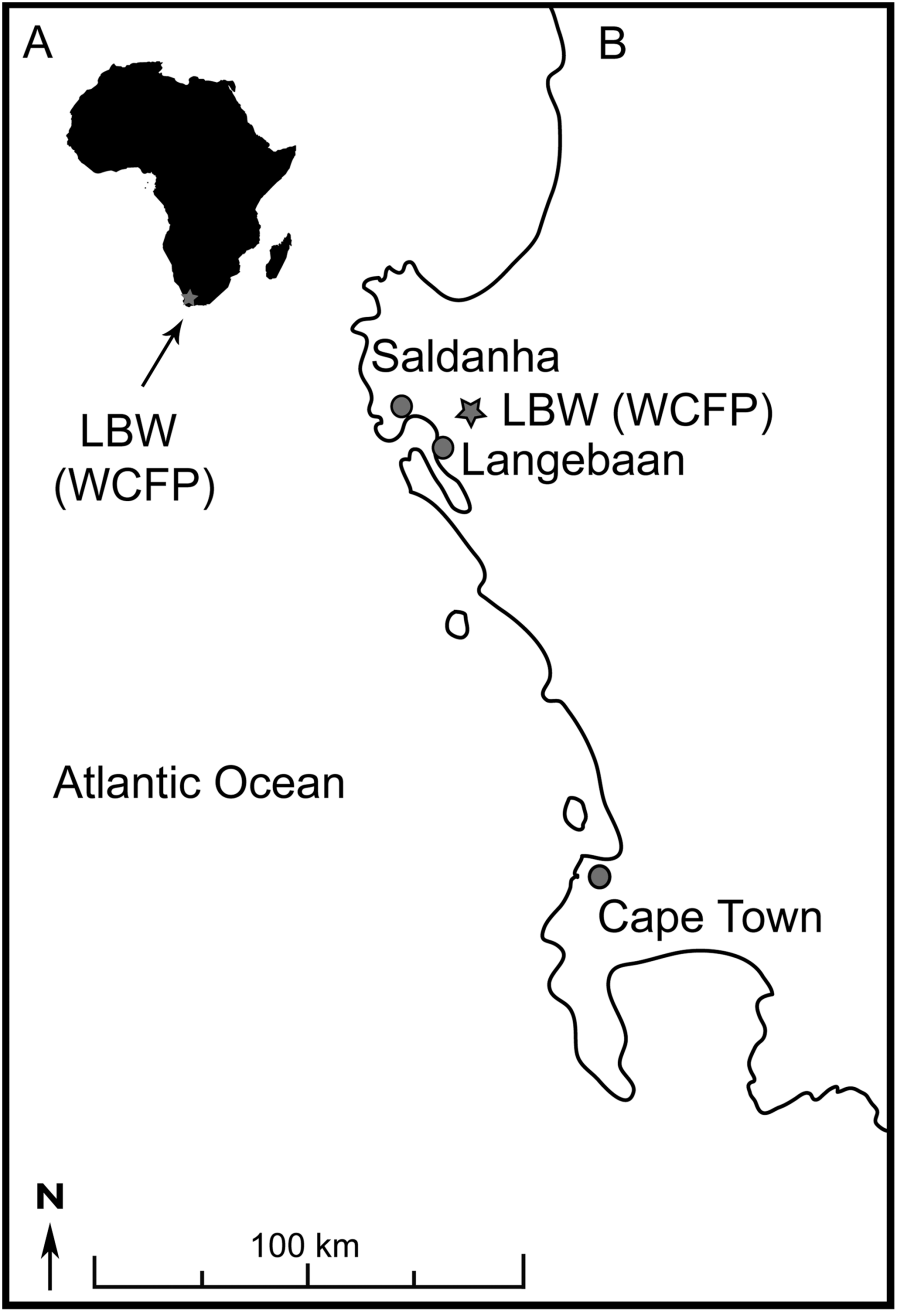
Location of Langebaanweg fossil site. (A) Silhouette of Africa, indicating the situation of Langebaanweg (gray star). (B) Simplified geographic map of South Africa. WCFP, West Coast Fossil Park.

Langebaanweg (LBW), ‘E’ Quarry, (a late Miocene—early Pliocene fossil site) has yielded one of the richest and best-preserved Neogene mammal assemblages in Africa (Hendey, 1981a; Hendey, 1982; Werdelin & Peigné, 2010). It is located within the West Coast Fossil Park, southwestern Cape, Langebaan (South Africa) (Fig. 1). The fossils occur in the Varswater Fm., which is divided in four members with different age, spatial relationships, thickness, lithology, and depositional setting (Roberts et al., 2011). Langeberg Quartz Sand Member (LQSM) and Muishond Fontein Pelletal Phosphorite Members (MPPM) represent the main fossil bearing deposits within the formations (Hendey, 1974, 1976, 1978a, 1978b, 1980, 1982; Roberts, 2006; Roberts et al., 2011). The MPPM has two different fossiliferous beds, Beds 3aN and Bed 3aS. These are interpreted as river channel deposits (Hendey, 1982), and inferred as being close in age, Bed 3aS somewhat older (Hendey, 1981b). Estimations based on paleomagnetic data and global sea level reconstructions indicate a similar age of ~5.15 ± 0.1 Ma for both LQSM and MPPM, suggesting that the fossils accumulated at an early stage in the Early Pliocene transgression (Roberts et al., 2011).

Carnivorans from LBW are quite common in the locality and have become a reference for Mio/Pliocene studies of taxonomy, systematic and paleobiology (Hendey, 1972, 1974, 1976, 1978a, 1978b, 1980, 1981a, 1982; Werdelin, Turner & Solounias, 1994; Werdelin & Lewis, 2001; Morales, Pickford & Soria, 2005; Morales & Pickford, 2005; Werdelin, 2006; Werdelin & Sardella, 2007; Stynder, 2009; Govender, Avery & Chinsamy, 2011; Tseng & Stynder, 2011; Govender, Chinsamy & Ackermann, 2012; Oldfield et al., 2012; Stynder et al., 2012, 2018; Stynder & Kupczik, 2013; Hartstone-Rose & Stynder, 2013; Govender, 2015; Hartstone-Rose et al., 2016). It contains a combination of archaic Miocene carnivorans and derived Pliocene ones, as befits its temporal position at the Miocene–Pliocene boundary and its geographic location at the southern tip of the continent (Werdelin, 2006). Among these there are two large mustelids, *Sivaonyx* Pilgrim, 1931 (previously determined as *Enhydriodon* Falconer, 1868) and *Plesiogulo* Zdansky, 1924, that can be classified as giant mustelids. Gigantism in mustelids appears early in their evolutionary history, as observed in several independent radiations in North America, Eurasia and Africa throughout the Neogene and Quaternary, and has developed in different subfamilies through the Miocene and Pliocene (e.g., Harrison, 1981; Werdelin, 2003a; Geraads et al., 2011; Wolsan & Sotnikova, 2013; Valenciano et al., 2015, Valenciano et al., 2016, Valenciano et al., 2017a, 2017b, Valenciano et al., 2020). The definition of a giant mustelids was provided by Werdelin (2003a), who stated that were extinct mustelids with an estimated mass more than twice that of the largest living forms. The African fossil record of giant mustelids includes relatives of living otters, wolverines and honey badgers. The giant otters are a diverse group of large to very large-sized species from the late Miocene to the early Pleistocene, represented by genera *Enhydriodon*, and *Sivaonyx* (Stromer, 1920, 1931; Hendey, 1974, 1978b; Petter, Pickford & Howell, 1991; Petter, 1994; Werdelin, 2003b; Morales & Pickford, 2005; Morales, Pickford & Soria, 2005; Pickford, 2007; De Bonis et al., 2008; Haile-Selassie, 2008; Peigné et al., 2008; Lewis, 2008; Haile-Selassie & Howell, 2009; Werdelin & Peigné, 2010; Geraads et al., 2011; Werdelin & Manthi, 2012; Grohé et al., 2013; Werdelin, Lewis & Haile-Selassie, 2014; Werdelin & Lewis, 2013; Werdelin & Lewis, 2017). Other large African mustelids are *Plesiogulo*, a large sized relative of the living wolverine (*Gulo gulo* Linnaeus, 1758) found in the Mio-Pliocene (Haile-Selassie, Hlusko & Howell, 2004; Hendey, 1978b; Morales, Pickford & Soria, 2005; Morales, Pickford & Valenciano, 2016), and the late Miocene cursorial *Ekorus ekakeran* Werdelin (2003a), a relative of the living honey badger *Mellivora capensis* (Schreber, 1776) (Valenciano et al., 2017b, 2020).

Herein, we present new fossils and a detailed review of the previously known material of the large mustelids *Sivaonyx hendeyi* (Morales, Pickford & Soria, 2005) and *Plesiogulo* aff. *monspessulanus* Viret (1939) from LBW housed at ISAM, in order to update our knowledge of this significant guild of large carnivores.

## Materials and Methods

### Nomenclature and measurements

**Figure 2:**
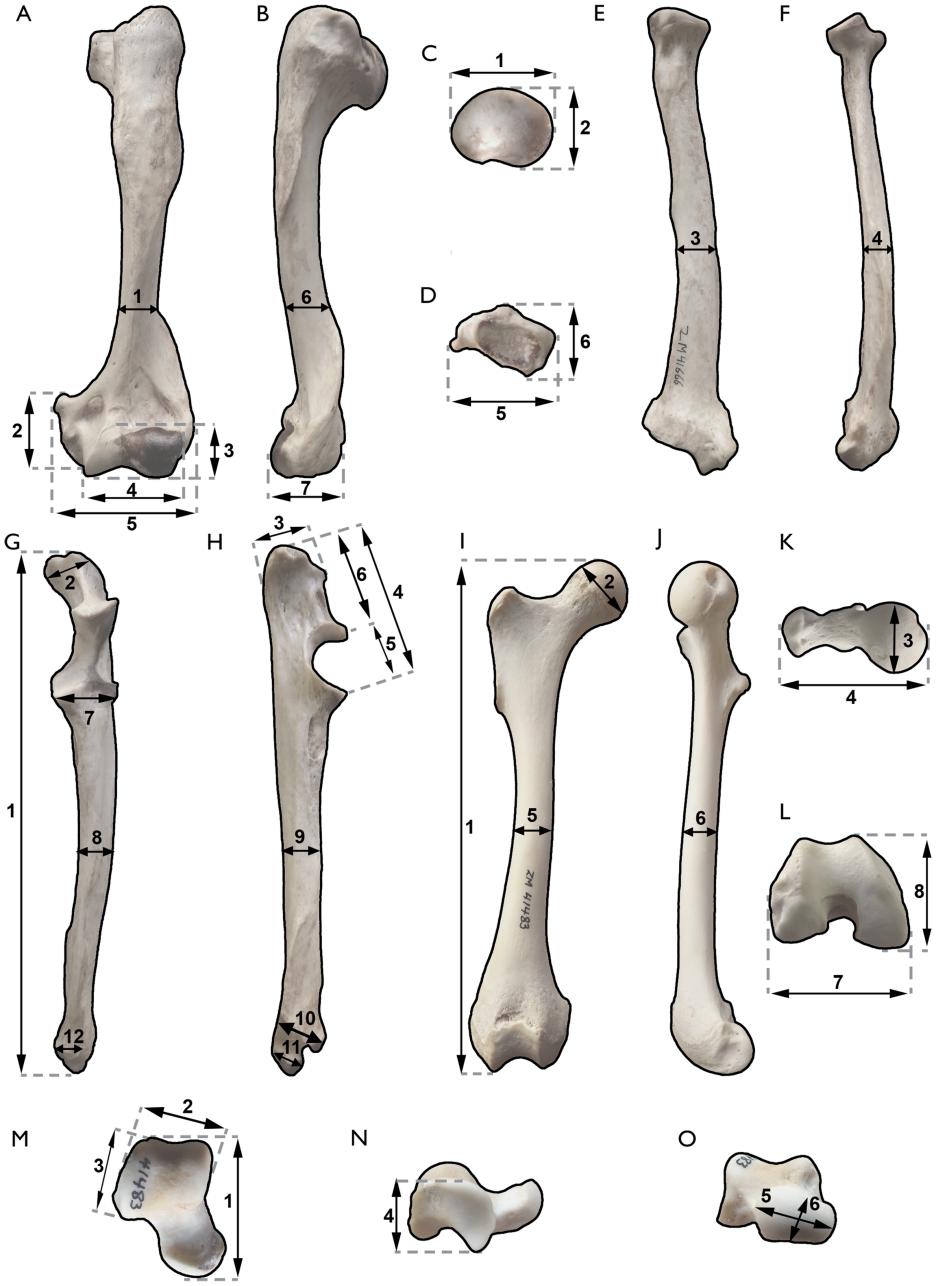
Postcranial measurements used in this work. (A and B) humerus. (A) cranial, and (B), lateral views. (C–F) radius. (C) proximal, (D) distal, (E) cranial, and (F) medial views. (G and H) ulna: (G) cranial, and (H), medial views; (I–L) femur: (I), cranial, (J), medial, (K), proximal, (L), distal views; (M–O) astragalus: (M), dorsal (N), lateral, and (O), distal views. Meaning of the measurements: humerus, 1, lateromedial width of the diaphyseal shaft measured at the last third of the bone, where the lateral crest of M. *Anconeus* finish, 2, height of the medial epicondyle, 3, (height) and 4, (length) of the humeral condyle, 5, maximum lateromedial width of the distal epiphysis, 6, craniocaudal width of the measure 1, and 7, craniocaudal width of the lateral epicondyle; Radius, 1, (lateromedial) and 2, (craniocaudal) widths of the proximal epiphysis, 3, (lateromedial) and 4, (craniocaudal) widths of the middle point of the diaphysis, 5, (lateromedial) and 6, (craniocaudal) widths of the distal epiphysis; Ulna, 1, total length, 2, maximum lateromedial width of the olecranon tuber, 3, maximum craniocaudal width of the olecranon tuber 4, proximodistal height of the proximal epiphysis of the ulna, measured from the proximal edge of the olecranon to the distal edge of the radial notch. 5, proximodistal height of the trochlear notch, 6, proximodistal height of the olecranon, 7, lateromedial width of the radial notch, comprising both medial and lateral coronoid processes, 8 (lateromedial) and 9 (craniocaudal) widths of the middle point of the diaphysis, 10, craniocaudal width of the distal epiphysis at the level of the articular circumference, 11 (craniocaudal) and 12 (lateromedial) widths of the styloid process; Femur, 1, total length, 2, lateroproximal-mediodistal width of the articular head, 3, craniocaudal width of the articular head, 4, maximum lateromedial width of the proximal epiphysis, 5, (lateromedial) and 6, (craniocaudal) widths of the middle point of the diaphysis, 7, lateromedial width of the distal epiphysis, and 8, craniocaudal length of the distal epiphysis; Astragalus, 1, total length, 2, (mediolateral width) and 3, (proximodistal length) of the trochlea, 4, maximum height of the tarsal, 5, (lateromedial) and 6, (dorsoplantar) widths of the head.

Dental nomenclature follows Ginsburg (1999) and Smith & Dodson (2003). Anatomical descriptions are based primarily on Barone (1999, 2000), Waibl et al. (2005), Evans & De Lahunta (2010, 2013) and Ercoli et al. (2013, 2015). The terminology conforms to the standard of the *Nomina Anatomica Veterinaria* (NAV; Waibl et al., 2005). Measurements were taken using Mitutoyo Absolute digital calipers to the nearest 0.1 mm (Tables 1–5; Fig. 2).

**Table 1:** Upper tooth measurements in mm of the new specimens of *Sivaonyx* and *Plesiogulo* from Langebaanweg (SAM-PQL), compared to other similar African species. L = length, W = width. Parenthesis means measurements on alveoli or at the base of the broken crown. *New measurement or re-measured after Hendey (1978b). Source: Morales & Pickford (2005), Haile-Selassie et al. (2004), Peigné et al. (2008), Geraads et al. (2011), Grohé et al. (2013), and this manuscript.

Taxa/Specimen	I1		I2		I3		P2		P3		P4		M1	
	L	W	L	W	L	W	L	W	L	W	L	W	L	W
*Sivaonyx hendeyi*														
SAM-PQL-52861								5.7						
SAM-PQL-50000C									9.7	8.1				
SAM-PQL-50000B*											16.9	17.4		
*Sivaonyx africanus*														
BSPG 1930 XI 1 (holotype)													14	18.7
*Sivaonyx beyi*														
TM 90-00-066													11.7	17.3
*Sivaonyx ekecaman*														
KNM-KP 10034 (holotype)													15.8	19.0
*Sivaonyx soriae*														
BAR 1982′01													12.3	(18)
BAR 1720′00											14.8	15		
*Enhydriodon dikikae*														
DIK-56-9 (holotype)									12.8	11.3	21	22.6	21.9	25.8
*Plesiogulo* aff. *monspessulanus*														
SAM-PQL-47086													(15.2)	(18.1)
SAM-PQL-40117									(13.9)	(9.5)	(22.9)	(14.8)	(12.5)	(17.9)
SAM-PQL-40042*			9.6	5.8			9.9	7.2	13.9	9.0	23.2	15.3		
SAM-PQL-21570*	6.3	3.6	10.8	6.1	12.6	10.5								
*Plesiogulo botori*														
KNM-NK-41420 (holotype)									14.4	10.2	24.5	16.7	15.9	21.2

**Table 2:** Lower tooth measurements in mm of the new specimens of *Sivaonyx* and *Plesiogulo* from Langebaanweg (SAM-PQL), compared to other similar African species. L = length, W = width. Parenthesis means measurements on alveoli or at the base of the broken crown. *New measurement or re-measured after Hendey (1978b). 1 = Venta del Moro locality, 2 = Las Casiones locality, 3 = Wikieup area locality. Sources: Schlosser (1903), Viret (1939), Kurtén (1970), Harrison (1981), Alcalá, Montoya & Morales (1994), Morales & Pickford (2005), Haile-Selassie et al. (2004), Peigné et al. (2008), Montoya, Morales & Abella (2011), Geraads et al. (2011), Grohé et al. (2013), and this manuscript.

Taxa/Specimen	p2		p3		p4		m1			m2	
	L	W	L	W	L	W	L	W	Wtl	L	W
*Sivaonyx hendeyi*											
SAM-PQL-50000A (holotype)*	(5.0)	(4.2)	(7.7)	(5.2)	12.0	9.4	21.3	13.9		8.1	10.3
SAM-PQL-9138*			(7.8)	(4.6)	13.8	9.9	(22.1)	(12.8)		(9.2)	(7.2)
*Sivaonyx africanus*											
BSPG 1930 XI 1 (holotype)					11.7	8.6	22.1	12.6			
*Sivaonyx beyi*											
TM 171-01-033 (holotype)					12.4	9.5	20.3				
TM 172-05-001							22.8	13.4			
*TM 355-02-002*					12.2		20	11.6			
*TM 247-01-005*							21.5	12.7			
*Sivaonyx ekecaman*											
KNM-KP 10034 (holotype)							21.2	13.5			
BAR 567′05							20.1	13			
BAR 720′03					11.3	8.4		12.8			
*Sivaonyx soriae*											
KNM-LU 337 & 338 (holotype)							17.6	10.5			
BAR 1984’057							17.5	10.6			
*Sivaonyx kamuhangirei*											
Unnumbered (holotype)							26	15.9			
*Sivaonyx bathygnathus*											
GSI D 33 (holotype)							17.1	9.7			
*Djourabus dabba*											
TM 293-01-006 & 053 (holotype)							20.9	14.7			
*Enhydriodon dikikae*											
DIK-56-9 (holotype)					16.2	11.9	(30)	(20)			
DIK-24-1							26	16.2			
*Plesiogulo* aff. *monspessulanus*											
SAM-PQL-21570*	—	—	11.1	7.4	15.7	9.5	(28.3)	(10.0)	–	11.6	8.7
SAM-PQL-40042*	9.1	6.6	12.5	8.1	15.9	9.4	–	–	–	–	–
SAM-PQL-28394*	–	–	–	–	–	–	(27.2)	11.5	9.7	7.2	6.7
*Plesiogulo monspessulanus*											
FSL, 40187 (holotype)			10	7	14	9	28	10.5			
*Plesiogulo monspessulanus*^1^											
VV16615			12.1	9.1	16.1	11	33.3	(11)			
*Plesiogulo monspessulanus*^2^											
KS-3							28	11.5			
*Plesiogulo lindsayi*^3^											
F:AM 49369, type locality	8.1	6.3	11	6.9	15	9	26.1	10.7			
*Plesiogulo marshalli*											
KUVP-3463, holotype			10.6	8.4	13.2	9	24.3	10			
*Plesiogulo crassa*											
Licent Collection 10.261 (holotype)	7.2		9.9		12.5		23			6.2	
*Plesiogulo brachygnathus*											
Unnumbered (holotype)			8.3	4.5	9.6	5.4	17.3	7			

**Table 3:** Postcranial measurements in mm of the new specimens of *Sivaonyx hendeyi*, compared with other Mio-Pliocene and the extant otter Aonyx capensis. Measurements 1–3 and 6 for humerus, 2–12 for the ulna, 4 for the femur, and 2–6 for the astragalus of *S. beyi* taken from pictures of the original. The measurements of *Enhydriodon* sp., and *Torolutra ougandensis* from Middle Awash taken from the pictures of Werdelin, Lewis & Haile-Selassie (2014) and Haile-Selassie (2008) respectively. *New measurement or re-measured after Hendey (1978b). *Torolutra ougandensis* 1 from Middle Awash and 2 from Nkondo (Uganda). Measurement of the femur 9 = Femoral robustness index × 100 of Samuels, Meachen & Sakai (2013), and 10= Femoral epicondylar index × 100 of Samuels, Meachen & Sakai (2013). Sources: Peigné et al. (2008), Petter (1994), Geraads et al. (2011), Werdelin, Lewis & Haile-Selassie (2014), and this work.

Measurement	1	2	3	4	5	6	7	8	9	10	11	12
**Humerus**												
*Sivaonyx hendeyi* SAM-PQL-60416		23.2	17.5	29.8	45.0		21.8					
*Sivaonyx beyi* TM 171-01-033	22.0	27.0	18.0	36.7	53.3	21.1	24.5					
*Enhydriodon dikikae* DIK-78-1				57.0	80.0							
*Torolutra ougandensis*^1^ *STD-VP-1/2*	14.0	14.0	11.0	24.0	34.0							
*Torolutra ougandensis*^2^ *NK-528′86*					35.0		16.0					
*Enhydritherium terraenovae* UF100000	11.9			26.6								
*Satherium piscinarium* USNM 23266					33.4							
*Aonyx capensis* SAM-ZM-41474	8.9	13.7	11.1	19.8	31.7	13.7	14.4					
*Aonyx capensis* SAM-ZM-41533	8.5	12.9	11.0	18.9	32.0	12.9	13.8					
**Ulna**												
*Sivaonyx hendeyi* SAM-PQL-21264									16.6	21.0	11.5	7.7
*Sivaonyx beyi TM 171-01-033*	185.0	17.0	22.0	58.0	20.0	38.0	17.0	12.0	15.0	19.0	11.0	8.0
*Enhydritherium terraenovae* UF100000	116.7									16.0		
*Aonyx capensis* SAM-ZM-41474	107.7	12.1	15.8	30.7	15.0	15.8	14.5	6.2	10.0	11.3	6.6	5.3
**Femur**												
*Sivaonyx hendeyi* SAM-PQL-50120		21.9	21.5									
*Sivaonyx hendeyi* SAM-PQL-41523*	164.0	21.6	22.1	48.7	20.2	16.7	40.7	35.1	12.1	24.8		
*Sivaonyx beyi TM 171-01-033*	195.0			58.0	21.0	17.3			10.8			
*Enhydriodon dikikae* DIK-4-1	(270)						61.0					
*Enhydriodon dikikae* DIK-44-1				78.3			65.5			22.6		
*Enhydriodon dikikae* DIK-41-20						23.0						
*Enhydritherium terraenovae* UF100000	128.2				15.4		33.7		12.0	26.3		
*Satherium piscinarium* USNM 23266	101.7			31.5	12.6	11.4	34.0	29.4	12.4	33.4		
*Aonyx capensis* SAM-ZM-41474	110.0	14.6	15.7	34.6	11.1	10.4	29.8	25.0	10.1	27.1		
*Aonyx capensis* SAM-ZM-41533	112.0	15.2	15.8	33.5	10.8	10.2	28.3	25.5	9.6	25.2		
**Astragalus**												
Sivaonyx hendeyi SAM-PQL-72172	31.9	24.8	27.3	15.6	16.0	9.6						
*Sivaonyx beyi TM 171-01-033*	34.9	23.0	25.0	18.0	18.0	8.0						
*Enhydriodon* sp. MDS-VP-3/19	42.0	26.0	31.0		24.0							
*Enhydritherium terraenovae* UF100000	23.8											
*Satherium piscinarium* USNM 23266	25.5											
*Aonyx capensis* SAM-ZM-41474	24.2	11.9	15.2	12.2	11.8	7.3						

**Table 4:** Radius measurements (in mm) of SAM-PQL-50001A and SAM-PQL-50001B compared with other living and extinct carnivorans.

Measurements	1	2	3	4	5	6	7
cf. *Viverra leakeyi* SAM-PQL-50001A						23.0	19.9
cf. *Viverra leakeyi* SAM-PQL-50001B						23.6	19.7
*Aonyx capensis* SAM-ZM-41474	81.1	13.2	9.5	6.6	6.4	17.4	12.8
*Viverra leakeyi* SAM-PQL-22061	157.9	15.4	11.4	11.2	7.1	21.9	17.9

**Note:**

Measure 1 = maximum length of the radius; 2–7 = measurements 1–6 of Fig. 2.

**Table 5:** Postcranial measurements in mm of the new specimens of *Plesiogulo* aff. *monspessulanus*, compared with all the measurement published of *Plesiogulo* spp., and the living wolverine (*Gulo gulo*). *New measurement or re-measured after Hendey (1978b). Measurement of the ulna 13 = Olecranon length index × 100 of Samuels, Meachen & Sakai (2013), and 14 = Ulnar robustness index × 100 of Samuels, Meachen & Sakai (2013).

Measurement	1	2	3	4	5	6	7	8	9	10	11	12	13	14
**Humerus**														
*Plesiogulo* aff. *monspessulanus SAM-PQL-6246*	16.5		21.3	27.7	(44.4)	18.2	36.9							
*Plesiogulo* aff. monspessulanus SAM-PQL-40042*	16.2	29.2	25.3	35.2	54.5	20.8	34.2							
*Plesiogulo marshalli* F:AM 108052	14.0	18.5	18.3	28.0	37.5	15.0	27.5							
*Plesiogulo marshalli* F:AM 67650A	14.5		18.5	25	(30.0)	15.0	29.0							
*Gulo gulo* NRM 20115498	11.1	16.4	15.7	26.1	41.1	13.3								
*Gulo gulo* FMNH-151027	14.2	20.7	19.2	27.8	41.1	14.5								
*Gulo gulo* FMNH-129317	12.0	17.7	13.3	22.3	34.4	10.6								
**Radius**														
*Plesiogulo* aff. *monspessulanus SAM-PQL-3440*			14.0	11.9	29.3	20.1								
*Plesiogulo* aff. monspessulanus SAM-PQL-40042*	23.6	14.5	13.8	16.0	33.8	21.8								
*Gulo gulo* NRM 20115498	18.1	11.8	9.0	8.0	24.1	15.4								
*Gulo gulo* FMNH-151027	17.4	11.8	8.8	7.5	23.4	14.2								
*Gulo gulo* FMNH-129317	17.4	10.9	6.9	6.2	21.2	13.7								
**Ulna**														
*Plesiogulo* aff. *monspessulanus SAM-PQL-36414*							(20.4)	16.0	(20.4)					
*Plesiogulo* aff. monspessulanus SAM-PQL-40042*	184.0	22.0	30.8	60.0	28.9	33.7	(22.5)	15.2	19.0	21.2	17.7	11.5	22.4	10.1
*Plesiogulo lindsayi* F:AM 108060	158.1	(25.5)							21.0	19.5	14.5			
*Plesiogulo marshalli* F:AM 108052	149.6		17.4	40.0	23.0	24.6			12.5	17.5	10.0		19.7	
*Gulo gulo* NRM 20115498	149.5	13.1	19.0	34.3	17.0	18.2	18.0	9.7	13.0	14.86			13.9	7.4
*Gulo gulo* FMNH-151027	143.0	14.5	20.2	32.4	14.3	20.7	16.5	9.6	11.8	14.9			16.9	7.8
*Gulo gulo* FMNH-129317	131.8	13.6	15.3	30.0	16.7	15.8	12.0	5.8	8.8	14.1			18.8	6.9

### Study material

We have re-analysed the mustelid material of *Sivaonyx* and *Plesiogulo* described by Hendey (1974, 1978b) such as new one housed in the Cenozoic collections at the Iziko South African Museum (ISAM). The comparative material of large Miocene mustelids consists of the following taxa: original mandible and skull UF100000 of *Enhydritherium terraenovae* Berta & Morgan (1985) from The Moss Acres Racetrack site (Florida, USA), and cast of the postcranial skeleton of the same specimen housed at UF. Cast of the holotype of *Sivaonyx africanus* (Stromer, 1931) housed at UF and pictures of the holotype housed at BSPG. Cast of both *Sivaonyx ekecaman* Werdelin (2003b), and *Sivaonyx soriae* Morales & Pickford (2005) from Lukeino and Sagatia localities in Kenya housed at MNCN. Pictures of the postcranial skeleton of *Sivaonyx beyi* Peigné et al. (2008) from TM 219 (Toros-Menalla fossiliferous area, Chad), and the postcranial of *Enhydriodon dikikae* Geraads et al. (2011) from DIK-56, Dikika research area, Ethiopia. Furthermore, original fossils of *Plesiogulo crassa* Teilhard de Chardin (1945), from localities 30, 108, and 111 from China (Kurtén, 1970), housed at PMU; *Plesiogulo monspessulanus* Viret (1939), from Venta del Moro and Las Casiones, Spain, housed at MGUV and FCPT respectively; holotype of *Plesiogulo praecocidens* Kurtén (1970) from locality 45 from China; holotype of *Plesiogulo lindsayi* Harrison (1981), from Wikieup, and other localities such as Old Cabin Quarry, and Redington Quarry in Arizona, USA, housed at AMNH; and *Plesiogulo marshalli* (Martin, 1928) from Edson Quarry in Kansas, USA, Optima in Oklahoma, USA, Coffee Ranch in Texas, USA, Modesto reservoir in California, USA, San Juan Quarry in New Mexico, USA, and Boney Valley in Florida, USA housed at AMNH. The holotypes of *Plesiogulo monspessulanus* Viret (1939), from Montpellier, France, housed at FSL and *Plesiogulo botori* Haile-Selassie, Hlusko & Howell (2004) from Narok locality, Lemudong’o, Kenia housed at KNM were studied via pictures of the originals. The extant specimens analyzed in this paper are: the African clawless otter *Aonyx capensis* (Schinz, 1821) (SAM-ZM-41474, 41483); the wolverine *Gulo gulo* Linnaeus (1758) (MNCN-16748; USNM 275160, 272316, A06231, 265649, 242705, 108654, 096147; NRM-A825005, A845012, 20055154, 20115498, A815010, A587719, A885007, A795005, A825004; FMNH-151027, 129317); and the honey badger *Mellivora capensis* (SAM-ZM-41483, 41666).

## Systematic Paleontology

Order Carnivora Bowdich, 1821

Suborder Caniformia Kretzoi, 1943

Family Mustelidae Fischer von Waldheim, 1817

Subfamily Lutrinae Bonaparte, 1838

Genus *Sivaonyx* Pilgrim, 1931

**Type species:** *Sivaonyx bathygnathus* (Lydekker, 1884) by original designation.

**Other included species:** *S. africanus* (Stromer, 1931); *S. beyi*; *S. ekecaman* Werdelin, 2003b; *S. hendeyi*; *S. kamuhangirei* Morales & Pickford, 2005; *S. soriae* Morales & Pickford, 2005 (=*S. senutae* Morales & Pickford, 2005 following Peigné et al., 2008); *S. hessicus* (Lydekker, 1890); *Sivaonyx gandakasensis* Pickford, 2007.

**Remarks:** *Sivaonyx* and *Enhydriodon* represent the largest African genera of bunodont otters, and their systematic position are debated (Morales & Pickford, 2005; Geraads et al., 2011; Grohé et al., 2013; Werdelin & Lewis, 2013, 2017; Werdelin, 2015; Ghaffar & Akhtar, 2016). Morales & Pickford, 2005 reassigned most of the African specimens with available dentition from *Enhydriodon* to *Sivaonyx*, a suggestion followed later by many authors (Pickford, 2007; Peigné et al., 2008; Lewis, 2008; Haile-Selassie, 2008; Haile-Selassie & Howell, 2009; Werdelin & Peigné, 2010; Grohé et al., 2013; Koufos, Mayda & Kaya, 2018), although recently new findings questioned this proposal (Geraads et al., 2011; Werdelin & Lewis, 2013; Werdelin, 2015). The aim of this work is not to resolve this controversy, and below we refer these taxa following the proposal of Morales & Pickford (2005). We also accept the presence of very large *Enhydriodon* in Africa with *E. dikikae* and *Enhydriodon* sp. from Woranso-Mille Area, Afar Region, Ethiopia, 3.6 Ma (Werdelin, Lewis & Haile-Selassie, 2014).

*Sivaonyx hendeyi* (Morales, Pickford & Soria, 2005)

1974 *Enhydriodon africanus:* Hendey, p. 72, fig. 7.

1978b *Enhydriodon africanus:* Hendey, p. 349, figs. 9, 10, 11.

2005 *Enhydriodon hendeyi:* Morales, Pickford & Soria, p. 56, fig 6L.

**Holotype:** SAM-PQL-50000A, a left hemimandible with p2-3 alveoli and complete p4-m2 figured by Hendey, 1978a, fig.9.

**Type Locality:** Langebaanweg, MPPM (Langebaan, South Africa), early Pliocene ca., 5.2 Ma.

**Referred material:** SAM-PQL-9138, right hemimandible with alveoli for c, p2-3 and m2, and a broken p4 and m1; SAM-PQL-50000B, left P4; SAM-PQL-41523, left femur.

**New material from Langebaanweg (LQSM and MPPM, see Table 6):** SAM-PQL-72229, fragmented left I2?; SAM-PQL-69635, fragmented right I3?; SAM-PQL-52861, left fragmentary P2; SAM-PQL-50000C, right P3; SAM-PQL-60416, right distal humerus epiphysis; SAM-PQL-21264, half diaphysis and distal epiphysis of a left ulna; SAM-PQL-50120, left proximal epiphysis of a femur; SAM-PQL-72172, left astragalus.

**Diagnosis:** In Morales, Pickford & Soria, 2005.

**Table 6:** Location of *Sivaonyx hendeyi* and *Plesiogulo* aff. *monspessulanus* from Langebaanweg, including units, beds and horizonts.

Taxa	Specimen	LQSM	MPPM	Origin
*Sivaonyx hendeyi*	SAM-PQL-9138		x	3aS
	SAM-PQL-50000A		x	3aN
	SAM-PQL-50000B		x	3aN
	SAM-PQL-41523		x	3aS
	SAM-PQL-50117		x	3aN
	SAM-PQL-50000C		x	3aN
	SAM-PQL-52861	–	–	No information
	SAM-PQL-69635a		x	Bed 3aN, Dump 10
	SAM-PQL-69635b		x	Bed 3aN, Dump 10
	SAM-PQL-50120		x	Bed 3aN
	SAM-PQL-60416		x	W. Wall IWRP 1976/2 S. end
	SAM-PQL-21264	x		
	SAM-PQL-72172	–	–	No information
*Plesiogulo* aff. *mospessulanus*	SAM-PQL-21570	x		
	SAM-PQL-28394	x		
	SAM-PQL-40042		x	BCWW S. of T2-gray sand
	SAM-PQL-47086		x	W. Wall IWRP 1976/2 G5
	SAM-PQL-40117	?	?	Scrubber
	SAM-PQL-6246	–	–	No information
	SAM-PQL-3440		x	Bed 3aN
	SAM-PQL-6414	–	–	No information

**Emended Diagnosis:** Modified after Morales & Pickford (2005). *Sivaonyx* of medium to large size. Robust P3 with distal accessory cusp. P4 with subquadrate outline. Paracone-metastyle compressed transversely, with a residual notch between them. Parastyle of medium size. Buccal cingulum strong joining the metastyle and parastyle. Protocone lingually projected from the paracone but joined to it by a crista oblique which joins the lingual crest of the paracone. Mesial valley present, but of modest dimensions. The hypocone is low and extensive, connecting the protocone and closing the tooth lingually. The median valley of the tooth is wide. p4 with very robust and high posterior cuspid located in a buccal position, with a small lingual platform. m1robust, with a crescentic-shape paraconid, mesiolingually located. Metaconid higher than the paraconid. Protoconulid very well developed. Talonid short and very wide, dominated by an extensive but relatively low hypoconid.

**Differential Diagnosis:** Differs from *S. bathygnatus* in a larger size, and a more bunodont dentition. P4 with larger parastyle, and shorter paracone-metastyle edge, and less conical hypocone. Distal accessory cuspid of p4 more robust, with m1 less basined talonid and absence of hypoconulid; Differs from *S. africanus* in having a more developed p3, and more developed basal cingulum in p4-m1. m1 with lower height of trigonid and hypoconid, smaller protoconulid, and shallower talonid basin; Differs from *S. beyi* in having a more robust cingulum on p4. m1 more bunodont, with larger and more transversely orientated paraconid, more robust metaconid, and metaconid higher than paraconid. Smaller postcranial size, more robust femur comprising a thinner neck and a larger more proximally orientated head, less developed trochanters and less extended trochanteric fossa. Differs from *S. ekecaman* in a less robust P4 with smaller buccal cusps, and a less broad lingual platform in the distal part. m1 slenderer, with a more elongated paraconid, less conical metaconid, more robust hypoconid, a lesser development of the entoconid, entoconulid and a shallow talonid valley. Differs from *S. soriae* (*=S. senutae*) in a larger size and more robust cingulum, P4 without accessory cusp on the protocone, protocone more lingually projected, hypocone more mesiodistally extended, and less conical; m1 with paraconid more bucollingually wide, more robust metaconid, absent hypoconulid and shallower talonid basin. Differs from *S. kamuhangirei, Enhydriodon dikikae* and *Djourabus dabba* in smaller size. Differs from *S. kamuhangirei* in a higher hypoconid and a deeper talonid valley. Differs from *E. dikikae* in a slenderer mandibular corpus, P4 less robust, without accessory cusps on protocone, less conical protocone and hypocone. Shorter distal accessory cuspid of p4. m1 with lower trigonid cuspids, hypoconid more developed, and more reduced lingual cuspids on the talonid. Slenderer humerus and femur. Differs from *D. dabba* in a slenderer mandibular corpus, m1 more elongated and a less broad trigonid cuspids, paraconid with a lesser transverse orientation, and longer talonid with more robust cingulid and a non-reduced valley.

**Description**

**Figure 3:**
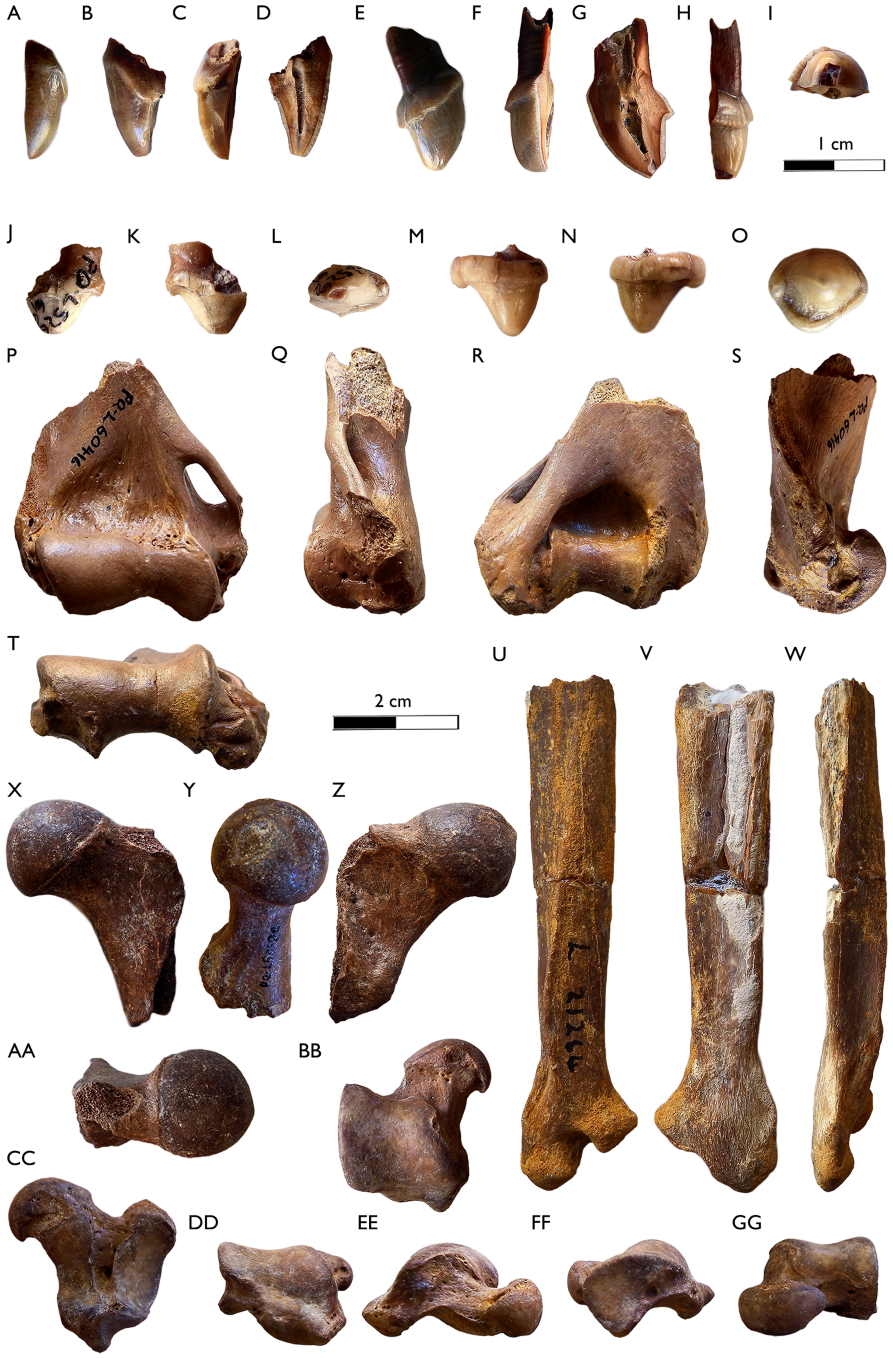
New dental and postcranial remains of *Sivaonyx hendeyi* from Langebaanweg (South Africa). (A–D) SAM-PQL-72172, left I2? fragment. (A) Buccal, (B) distal, (C) lingual, and (D) mesial view. (E–I) SAM-PQL-69635 right I3? fragment. (E) Buccal, (F) mesial, (G) lingual, (H) distal, and (I) oclussal views. (J–L) SAM-PQL-52861, left P2, fragment. (J) Buccal, (K) lingual, and (L) occlusal views. (M–O) SAM-PQL-50000C, right P3. (M) Buccal, (N) Lingual, and (O) occlusal view. (P–T) SAM-PQL-60416, right distal epiphysis of the humerus. (P) Rostral, (Q) medial, (R) caudal, (S) lateral, and (T) distal views. (U–W) SAM-PQL-21264, left diaphysis and distal epiphysis of the ulna. (U) Lateral view, (V) medial, and (W) caudal views. (X–AA) SAM-PQL-50120, left proximal femur. (X) Rostral, (Y) medial, caudal (Z) and (AA) proximl views. (BB–GG) SAM-PQL-72172, left astragalus. (BB) Dorsal, (CC) ventral, (DD) proximal, (EE) medial, (FF) lateral, and (GG) distal views. The scale bar of (A)–(O) equals 1 cm and (P)–(GG) equals 2 cm.

**SAM-PQL-72229:** It is a caniniform fragment of an upper incisor, tentatively determined as a left I2 (Figs. 3A–3D), with a single cusp lingually curved and a small wear facet on its tip. It represents the left half of the tooth. Its enamel is thick and wrinkled. There is a flat cingulum starting in the buccal part and running into the distal area, which is proximodistally enlarged. There is a crista from this point to the tip.

**SAM-PQL-69635:** It is a fragment of an indeterminate tooth, interpreted as a right I3 (Figs. 3E–3I). It is the right side of the tooth. Its shape suggests a conical crown. It is tall with a single cusp, lingually curved. As in SAM-PQL-72229, it has a wrinkled and thick enamel throughout the crown. A larger and crowned cingulum is present. There is a small projection of the cingulum in the lingual part. The tip has greater wear than those of the specimen SAM-PQL-72229.

**SAM-PQL-52861:** Left P2 mesially broken (Figs. 3J–3L; Table 1). It is elongated, oval in occlusal view, and unicuspid. It has two roots, but only the distal one is preserved. Its cusp is located in the mesial portion of the tooth. It is slightly worn. The tooth is mesially wider. There are crenulations and roughnes on the whole crown. There are also a sharp mesial and distal cristaes. A distally tall and crowned cingulum is present.

**SAM-PQL-50000C:** Complete right P3 (Figs. 3M–3O; Table 1). It is robust and bunodont with a lingual bulge. The main cusp is mesially located. There are two distal accessory cusps, a small one located on the most lingual point of the bulge and a larger one positioned on the distal corner of the premolar. The buccal wall is convex. A strong cingulum surrounds the whole crown, which displays a course texture.

**SAM-PQL-60416:** Distal epiphysis of a right humerus (Figs. 3P–3T; Table 3). The proximal part is broken. The distal epiphysis is broad and craniocaudally compressed. The preserved portion of the lateral epicondylar crest is laterally projected. There is a large surface area for the attachment of the M. *extensor carpi ulnaris* and the M. *extensor digitorum lateralis*. The trochlea and capitulum are relatively long in the lateromedial axis. Both radial and coronoid fossae are well development over the capitulum and trochlea respectively. The medial epicondyle is distomedially enlarged, increasing the surface area for the attachment of the M. *pronator teres* and the M. flexor *digitorum profundus*. A large and proximocaudally oval entepicondylar foramen is present. The olecranon fossa is deep. The supratrochlear foramen is absent.

**SAM-PQL-21264:** Fragmentary left ulna including the distal half portion of the diaphysis and the distal epiphysis (Figs. 3U–3W; Table 3). The lateral side is damaged, being proximodistally crushed and laterally collapsed. There is calcrete with yellow-orange iron minerals on the surface of the ulna, particularly in the cranial and medial faces. The interosseus tubercle is not preserved. Caudally, the ulna is sigmoid, with the medially projected crest for the attachment of the M. *pronator quadratus*. The distal epiphysis is robust, with a large and rostrally projected articular circumference and a large and round styloid process.

**SAM-PQL-50120:** Fragment of the left proximal femur. It is proximodistally broken (Figs. 3X–3AA; Table 3), and neither the trochanteric fossa nor the greater trochanter is preserved. The head is complete. It is round and the *fovea capitis* is located medially. The neck is short. In the caudal side, a robust lesser trochanter is caudomedially projected.

**SAM-PQL-72172:** Left astragalus (Figs. 3BB–3GG; Table 3). The body is trapezoidal in shape. There is a broad, shallow astragalar trochlea, and a noticeable medioproximal projection of the astragalar tubercle (plantar tendinal groove) (Fig. 3BB). It is a relatively robust, deep groove for the tendons of the plantar flexor muscles, and extends to the ventral side of the bone (Figs. 3CC and 3DD). The medial tibial facet is located on the medial portion of the trochlea that merges into the plantar tendinal groove (Fig. 3EE). The lateral area of the trochlea has a drop-shape fibular facet, slightly concave, and dorsoventrally higher (Fig. 3FF). In ventral view, the ectal facet is wider than the sustentacular facet. Both facets are separated by a deep groove, in which there are several foramina. The neck is relatively short, mediolaterally wide, and distomedially orientated (Figs. 3BB and 3GG). The head is lateromedially broad, dorsoventrally compressed and the navicular articular surface is strongly convex. It is orientated parallel to the mediolateral axis, with the lateral border being slightly more dorsally elevated to the medial one (Fig. 3GG).

**Discussion**

Large to giant otters were rather common at the end of the Miocene until the Pleistocene in Eurasia, North America and Africa (Berta & Morgan, 1985; Willemsen, 1992; Pickford, 2007; Werdelin & Peigné, 2010; Werdelin, 2015; Tseng et al., 2017). The group is represented by *Djourabus* Peigné et al., 2008, *Enhydriodon*, *Enhydritherium* Berta & Morgan, 1985, *Paludolutra* Hürzeler & Engesser, 1976, *Sivaonyx*, *Torolutra* Petter, Pickford & Howell, 1991, and *Vishnuonyx* Pilgrim, 1932. Apart from *Torolutra* and *Vishnuonyx*, these mustelids have a very robust dentition and are commonly known as bunodont otters. Some of them were the largest and most massive mustelids of all time, with estimated body masses exceeding 200 kg (e.g., *E. dikikae*; Geraads et al., 2011; Valenciano et al., 2017b). The preserved postcranial remains suggest an array of different lifestyles ranging from terrestrial to semi-aquatic (Lewis, 2008; Peigné et al., 2008; Geraads et al., 2011; Werdelin & Lewis, 2017). The phylogenetic relationships of these extinct otters are uncertain. In the single cladistics analysis performed so far (Wang et al., 2017), these mustelids constitute a paraphyletic clade related to the living *A. capensis* (the African clawless otter), *Lutra lutra* Linnaeus, 1758 (Eurasian otter) and *Enhydra lutris* Linnaeus, 1758 (sea otter).

**Figure 4:**
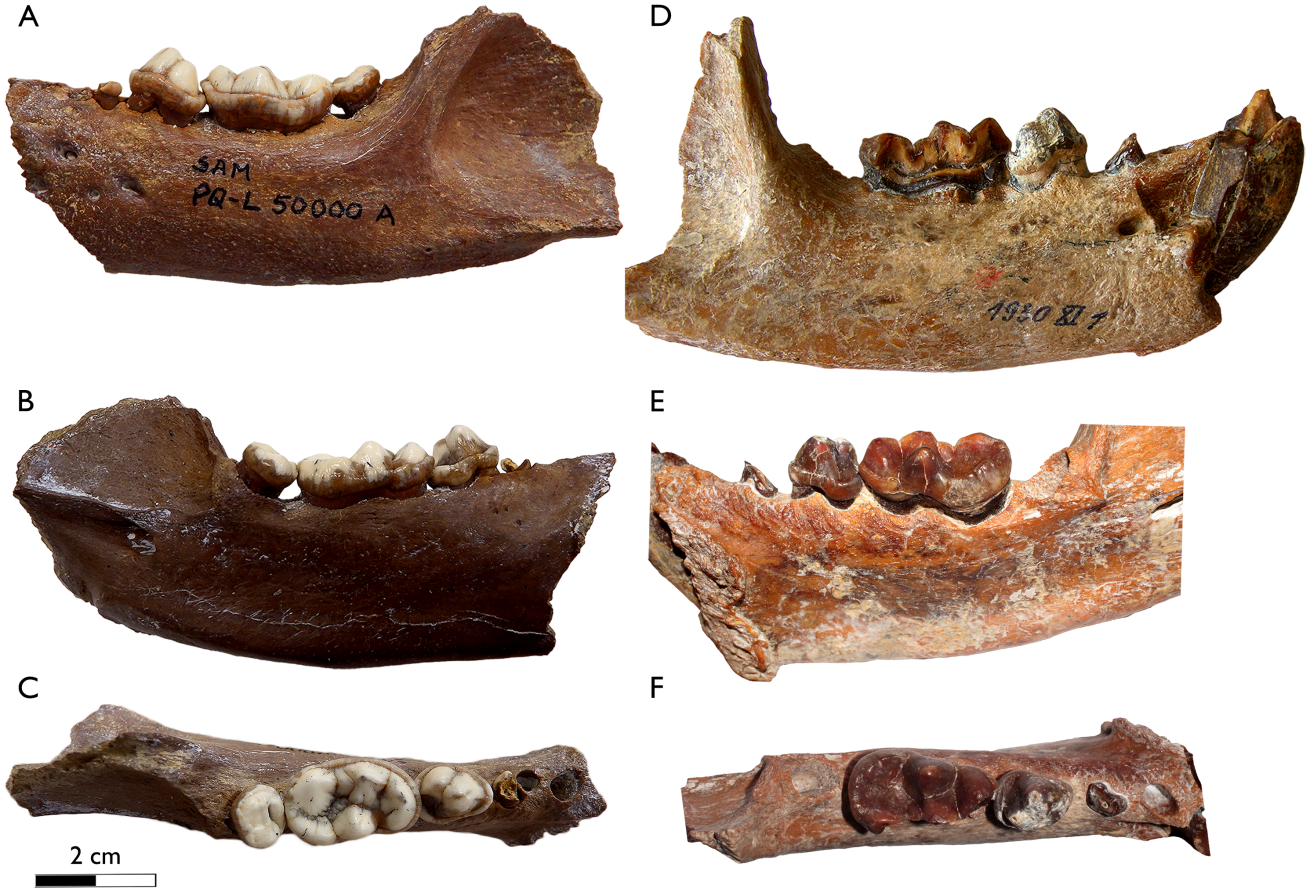
Comparison of the lower dentition of the Mio/Pliocene South African *Sivaonyx* spp. (A–C) SAM-PQL-50000A, left hemimandible, holotype of *Sivaonyx hendeyi* from Langebaanweg (South Africa). (A) Buccal, (B) lingual, and (C) occlusal views. (D–F) BSPG 1930 XI 1, right hemimandible, holotype of *Sivaonyx africanus* from Kleinzee (South Africa). (D) Buccal, (E) lingual and (F) occlusal view. The scale bar equals 2 cm.

Most of the African giant bunodont otters are represented by very scarce and fragmentary remains, which make any new fossils significant in order to understand the role and lifestyle of these peculiar mustelids. The first remains of an extinct large otter in South Africa was reported by Stromer (1931), who erected *Enhydriodon africanus* on the basis of a fragmentary right M1 and a right hemimandible with p3-m1 from the early Pliocene deposit of Klein Zee, Namaqualand, South Africa. A second discovery of the same taxon was reported by Hendey (1974, 1978b) in LBW, a similarly aged early Pliocene locality situated 500 km south of Klein Zee. He described new dental and postcranial material of *E. africanus* from the beds 3aN and 3aS of the MPPM, comprising two hemimandibles (SAM-PQL-9138 and SAM-PQL-50000A) and a left P4 (SAM-PQL-50000B), and tentatively assigned to this species a left femur (SAM-PQL-41523), two distal epiphysis of radii (SAM-PQL-50001A, B) and one astragalus (SAM-PQL-50117) (Hendey, 1978b). Morales, Pickford & Soria (2005) moved the LBW taxon to a new species, separate from Klein Zee, establishing *Enhydriodon hendeyi*, which included SAM-PQL-50000A (holotype), SAM-PQL-9138, SAM-PQL-50000B and SAM-PQL-41523. The astragalus SAM-PQL-50117 was re-interpreted as *Orycteropus* Geoffroy Saint Hilaire, 1796, a relative of the living aardvark (Pickford, 2005). The same year, Morales & Pickford (2005) reassigned the species from LBW and Klein Zee to the genus *Sivaonyx*, while retaining the species names. Despite the similar age and size, it is widely accepted that both South African bunodont otters are different species (Pickford, 2007; De Bonis et al., 2008; Haile-Selassie, 2008; Peigné et al., 2008; Lewis, 2008; Haile-Selassie & Howell, 2009; Werdelin & Peigné, 2010; Geraads et al., 2011; Grohé et al., 2013). *Sivaonyx hendeyi* can be distinguished from *S. africanus* (Fig. 4) in several dental traits summarized in the differential diagnosis of this manuscript. In general terms, *S. hendeyi* possesses a more robust dentition, including more robust cingulids, a more developed p3 and a m1 with lower crown, smaller protoconulid and shallower talonid valley to that of *S. africanus*. The new dental material of *S. hendeyi* described, include new data on the upper incisor and upper premolars, previously unknown. All the new dentition is robust and have strong cingula (Fig. 3), which are in consonance with the known robust and bunodont dentition (Hendey, 1974, 1978b).

The fragmentary nature of the preserved SAM-PQL-69635 (I3?) and SAM-PQL-72229 (I2?), make their anatomical determination problematic, and in this contribution are tentatively assigned to *S. hendeyi*. The presence of a comparable thick and deeply wrinkled enamel of the crown, in addition to the bunodont morphology of the I3 (SAM-PQL-69635), resembles the known dentition of this taxon. Incisors of bunodont otters are very scarce in the fossil record. The only upper incisors preserved are the I3 of the North American *En. terraenovae* from The Moss Acres Racetrack site (Florida, USA), Late Miocene (Hemphillian 2, c.a. 7–6 Ma) (Lambert, 1997), the Ethiopian *E. dikikae* from DIK-56 in the Dikika research area in Ethiopia (4–3.4 Ma) (Geraads et al., 2011) as well as the whole battery of upper incisors of the Chinese *Siamogale melilutra* Wang et al., 2017 from Shuitangba site in the south-west of China (late Miocene, ~6.2 Ma). The I3 (SAM-PQL-69635) is similar to the I3 of *E. dikikae* in being conical with a medial keel. The overall morphology of the P2 (SAM-PQL-52861), are very close to the one of *Si. melilutra* from Shuitangba (Wang et al., 2017). The P3 (SAM-PQL-50000C) of *S. hendeyi* also has the typical bunodont otter’s morphology, with a convex buccal wall, a lingual bulge and a robust cingulum surrounding the whole tooth, resembling those of *E. dikikae* and *En. terraenovae*; and unlike to those of *S. ekecaman* from Sagatia, Magabet Fm., Kenya which distal area is almost circular in occlusal outline and no distal accessory cusp is present (Morales, Pickford & Soria, 2005; Morales & Pickford, 2005). It is also dissimilar to *Si. melilutra* in having a wider P3 (Wang et al., 2017). The morphological differences of the dentition between *S. hendeyi* and the other African species of *Sivaonyx* (*S. ekecaman*, *S. soriae*, *S. beyi*, and *S. kamuhangirei*), as well as *En. dikikae* and *D. dabba* has been largely detailed (Werdelin, 2003b; Morales, Pickford & Soria, 2005; Morales & Pickford, 2005; Pickford, 2007; Peigné et al., 2008; Geraads et al., 2011) and is summarized in the differential diagnosis section. Metrically, the dentition of *S. hendeyi* is comparable to those of *S. africanus*, *S. ekecaman* from the Late Miocene-Early Pliocene of Ethiopia and Kenya (Werdelin, 2003b; Morales, Pickford & Soria, 2005; Morales & Pickford, 2005; Haile-Selassie, 2008), and *S. beyi* from Toros-Menalla fossiliferous area (Chad, c.a., 7 Ma) (Peigné et al., 2008) (Tables 1 and 2). All of them are larger than the type species of the genus *S. bathygnathus* from Hasnot, Punjab, Pakistan (Late Miocene) (Pickford, 2007) and also larger than the Kenyan and Ethiopian *S. soriae* (Morales & Pickford, 2005; Haile-Selassie, 2008) from the Late Miocene, but smaller than *Sivaonyx kamuhangirei* Morales & Pickford, 2005 from Kazinga, Uganda, c.a., 3.5 Ma (Pickford, 2007).

**Figure 5:**
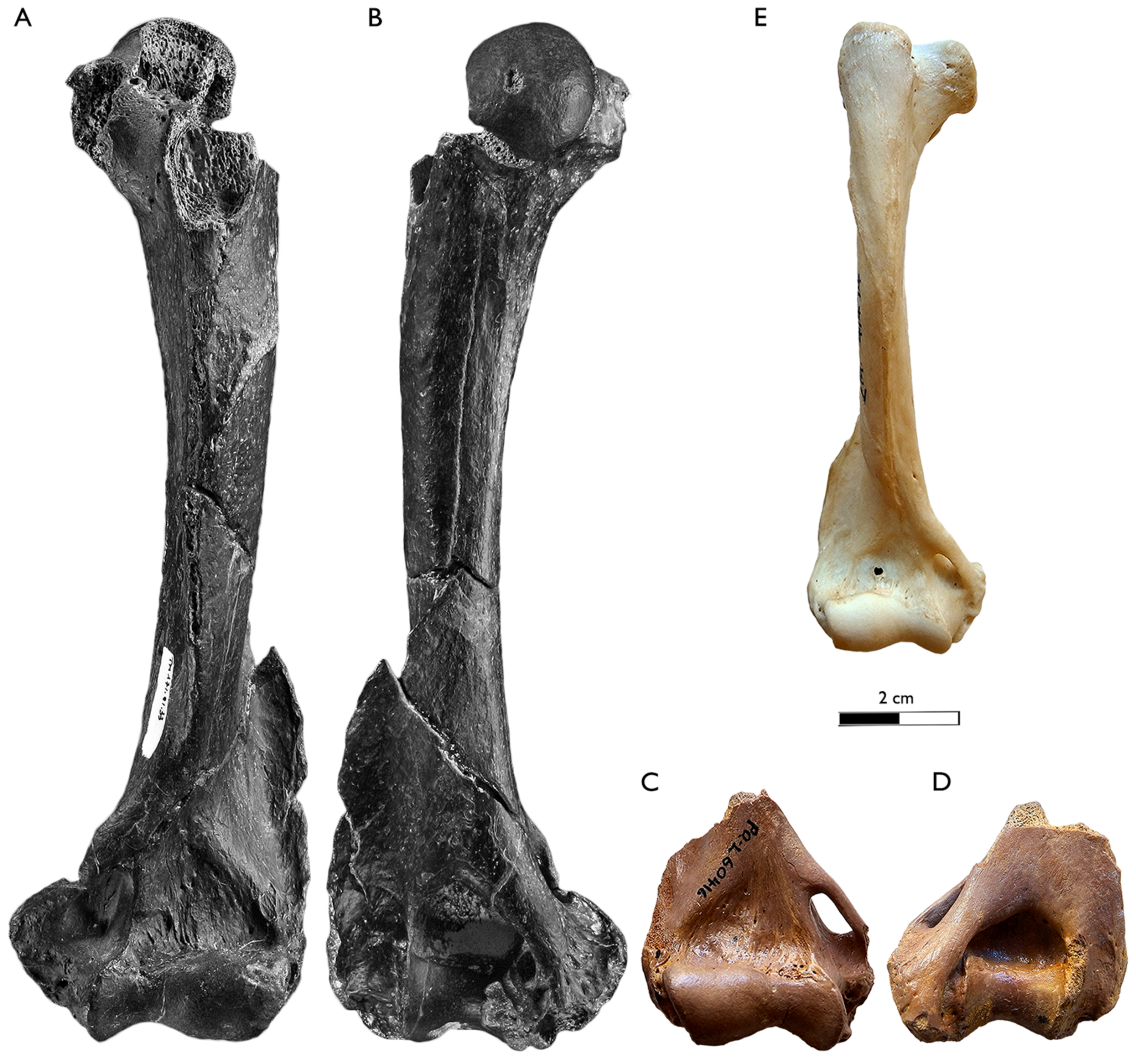
Comparison of the humerus of *Sivaonyx beyi* (Chad), *Sivaonyx hendeyi* (South Africa), and the extant African otter *Aonyx capensis*. (A and B) TM 171-01-033, part of the holotype of *Sivaonyx beyi* from TM 171, Toros-Menalla (Chad, Late Miocene), left humerus. (A) cranial, and (B) caudal views. (C and D) SAM-PQL-60416, right distal epiphysis of the humerus of *Sivaonyx hendeyi* from Langebaanweg (South Africa). (C) Cranial, and (D) caudal views. (E) ZM-4474, right humerus of *Aonyx capensis*. The scale bar equals 2 cm.

**Figure 6:**
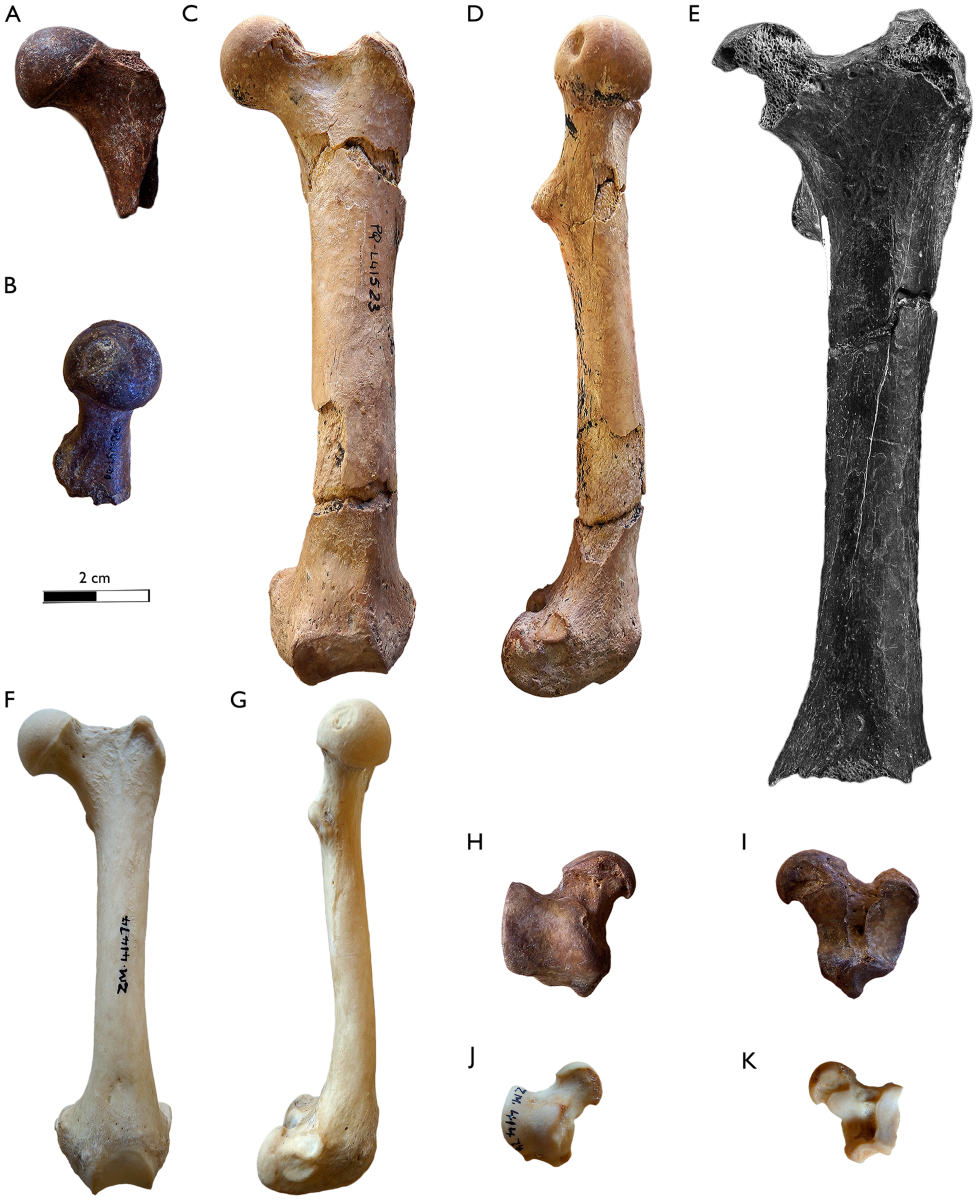
Comparison of the femur and astragalus of *Sivaonyx hendeyi* from *Langebaanweg* (South Africa), *Sivaonyx beyi* from TM 171, Toros-Menalla (Chad), and the extant African otter *Aonyx capensis*. (A and B) SAM-PQL-50120, left proximal epiphysis of the femur of *Sivaonyx hendeyi*. (A) rostral, and (B) medial views. (C and D) SAM-PQL-41523, left femur of *Sivaonyx hendeyi*. (C) Rostral, and (D) medial views. (E) TM 171-01-033, part of the holotype of *Sivaonyx beyi* from TM 171, fragment of left femur. (F and G) ZM-4474, left femur of *Aonyx capensis*. (F) Rostral, and (G) medial views. (H and I) SAM-PQL-72172, left astragalus of *Sivaonyx hendeyi*. (H). Dorsal, and (I) ventral views. (J and K) ZM-4474, left astragalus of *Aonyx capensis*. (J). Dorsal, and (K) ventral views. The scale bar equals 2 cm.

Postcranial remains of bunodont otters are even scarcer than craniodental material. With the exception of *S. beyi*, *S. hendeyi*, represents the only species of *Sivaonyx* for which postcranial bones have been described (Peigné et al., 2008). Additional large otters with postcranial bones recovered are *E. dikikae*, *Enhydriodon* sp., and *Torulutra ougandensis* Petter, Pickford & Howell, 1991 from Ethiopia and Uganda (Petter, 1994; Haile-Selassie, 2008; Geraads et al., 2011; Werdelin, Lewis & Haile-Selassie, 2014) as well as *En. terraenovae* from Florida (Lambert, 1997) and the late Pliocene *Satherium piscinarium* (Leidy, 1873) from the Hagerman fauna (Bjork, 1970), which was a large otter resembling the living South American *Pteronura* Gray, 1837, but with sharper dentition. The morphology of the fragmentary humerus SAM-PQL-60416 is similar to that of the *S. beyi* and the living *A. capensis* (Fig. 5). It shares with both lutrines the expanded medial epicondyle, which is more expanded in the Chadian one, and an enlarged lateral epicondylar crest. The dental proportions of *S. hendeyi* and *S. beyi* are analogous, but the postcranial skeleton of the former is larger (Figs. 5 and 6; Tables 2 and 3). The distal epiphysis of the humerus of *T. ougandensis* from Middle Awash and the North American *En. terraenovae* and *Sa. piscinarium* are similar, but smaller in size (Table 3). Moreover, the distal epiphysis SAM-PQL-60416 is rostrocaudally compressed, similar to *A. capensis* and *En. terraenovae* and unlike *E. dikikae*, which less rostrocaudal compression. The presence or lack of that trait in *S. beyi* was not described by Peigné et al. (2008). It is also differs from a distal epiphysis of a fragmentary humerus of a medium sized otter from Nkondo Uganda (5–4.5 Ma), determined as *Enhydriodon* sp. by Petter (1994, pl. 1, fig. 3-4). Later, Morales & Pickford (2005) transferred the dentition associated with this material to *S. kamuhangirei*, but they did not mention the humerus. Based on the relatively smaller dimensions of that humerus, Peigné et al. (2008) noted that it may belong to a smaller-sized species of *Sivaonyx* or to a different genus. We agree with their suggestion, and since its relatively small size compared to specimen SAM-PQL-60416, we discard its designation to *S. kamuhangirei* or *S. hendeyi*, being closer in shape and size to the medium size *T. ougandensis*, also present in Nkondo (Petter, Pickford & Howell, 1991). The fragmentary ulna (SAM-PQL-21264) is essentially identical to those of *S. beyi*, and shares traits with the semifossorial mustelid *M. capensis* (living honey badger). The distal area of the diaphysis of both *S. hendeyi* and *S. beyi* has a distinct medially projected crest for the attachment of the M. *pronator quadratus*. The articular circumference is robust and the rostrally projected styloid process is large and round. The ulna of *Sivaonyx* spp., is distinguished from semiaquatic otters such as the extant *A. capensis* and the extinct *En. terraenovae*. The former has a reduced crest, and a reduced articular process (Lambert, 1997).

Both preserved femora of *S. hendeyi* are of a similar size, while the proximal projection of the head of SAM-PQL-50120 is less than SAM-PQL-41523. The femora from LBW are smaller than those of *S. beyi*, and *E. dikikae* but larger than the extinct *En. terraenovae*, *Sa. piscinarium* and the living *A. capensis* (Fig. 6; Table 3). Following the sample and methodology of Samuels, Meachen & Sakai (2013), we calculated the femoral robustness index (FRI) and femoral epicondylar index (FEI) of *S. hendeyi* (SAM-PQL-41523), and the other extinct bunodont otters analyzed (Table 3), obtaining for both indices the highest values of the whole sample. The FRI value of *S. hendeyi* is similar to those of the extinct *S. beyi*, *En. Terraenovae*, and *Sa. piscinarium*, analogous to the largest living otters (*Enhydra* Fleming, 1822 and *Pteronura*), and higher to those of the living marine otter [*Lontra felina* (Molina, 1782)], smooth-coated otter [*Lutrogale perspicillata* (Geoffroy Saint Hilaire, 1826)], North American river otter [*Lontra canadensis* (Schreber, 1777)] and *A. capensis*, suggesting a possible allometric factor. The FEI value of *S. hendeyi* is analogous to the living African clawless otter and the Asian small-clawed otter [*Amblonyx cinereus* (Illiger, 1815)]. Interestingly, the lowest value of the analyzed extinct otters corresponds with the largest one, *E. dikikae*, with similar values to the semifossorial musteloids [American badger *Taxidea taxus* (Schreber, 1777), and the striped skunk *Mephitis mephitis* (Schreber, 1776)] and the generalist giant panda *Ailuropoda melanoleuca* (David, 1869). The highest value for that index was *Satherium*, which has values close to those of *Pteronura* and *Enhydra*. Semiaquatic carnivorans such as the lutrines are characterized by high FRI and FEI values, together with enlarged humeral epicondyles (Samuels, Meachen & Sakai, 2013; Fabre et al., 2015; Botton-Divet et al., 2016; Kilbourne & Hutchinson, 2019; Parsi-Pour & Killbourne, 2020). A robust femur indicates better abilities to resist bending and shearing stress, accordingly, the FRI is high in living semiaquatic and semifossorial carnivorans (Samuels, Meachen & Sakai, 2013; Fabre et al., 2015; Botton-Divet et al., 2016; Parsi-Pour & Killbourne, 2020). Higher FEI values indicate relatively large area available for the origins and insertion of the muscles *gastrocnemius* and *soleus*, used in extension of the knee and plantar-flexion of the pes (Samuels, Meachen & Sakai, 2013), with the lutrines having the highest values of the carnivorans. In the context of lutrinae, *Am. cinereus* and *A. capensis* are less linked to water bodies than other river otters and are interpreted by some authors as the least aquatic living otters (Perrin & Carugati, 2000a, 2000b; Angelici et al., 2005; Kruuk, 2006; Lewis, 2008).

**Figure 7:**
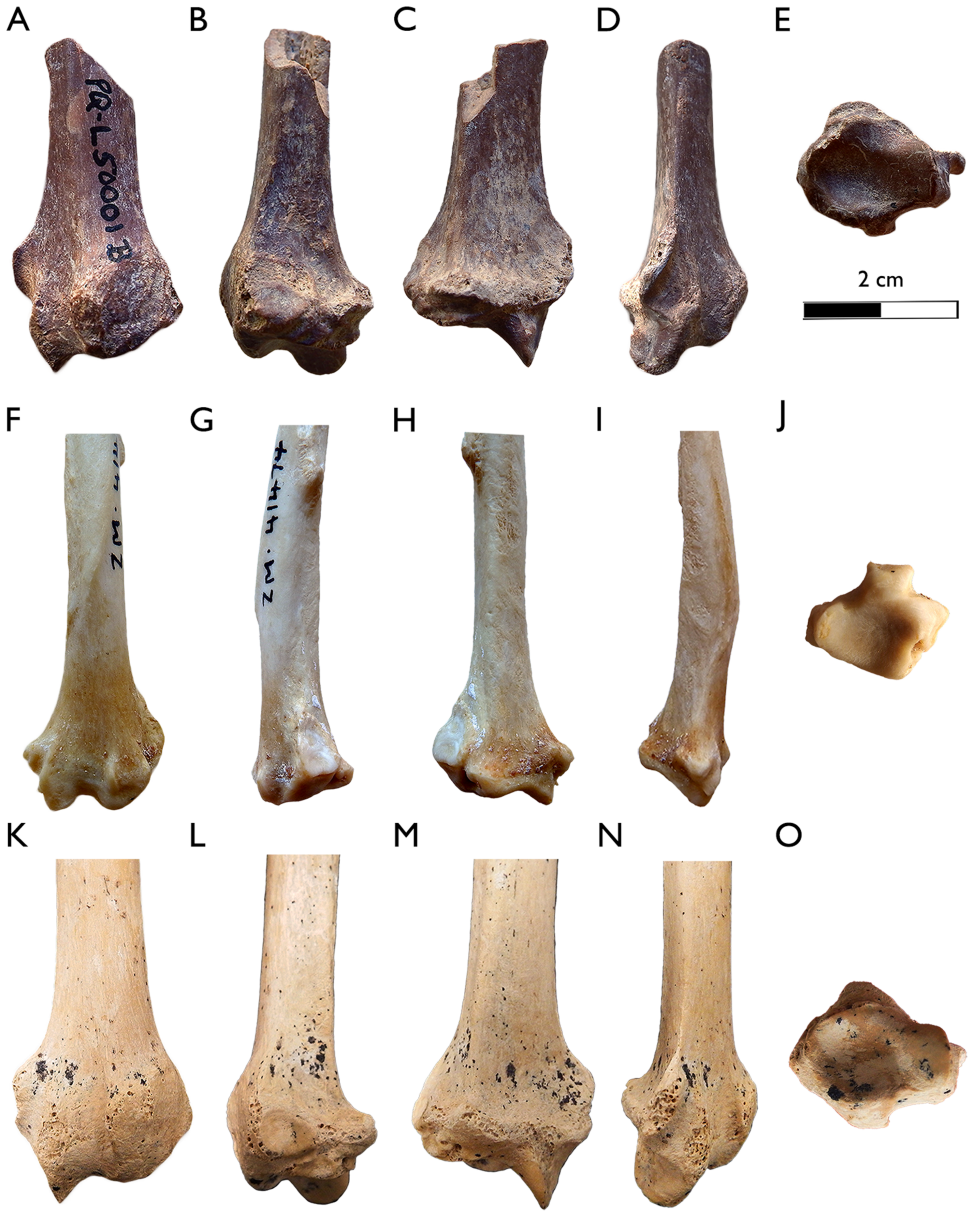
Comparison of the left distal epiphysis of the radius of the specimen SAM-PQL-50001B from Langebaanweg with other carnivorans. (A–E) SAM-PQL-50001B, herein reallocated to cf. *Viverra leakeyi*. (F–J) Left distal epiphysis of the radius of the living African clawless otter *Aonyx capensis*. (K–O) SAM-PQL-22061, left distal epiphysis of the radius of cf. *Viverra leakeyi* from Langebaanweg. (A), (F) and (K) cranial views; (B), (G) and (L) lateral views. (C), (H) and (M) caudal views. (D), (I) and (N) medial views. (E), (J) and (O) distal views. The scale bar equals 2 cm.

The astragalus (SAM-PQL-72172) of *S. hendeyi* is comparable in general morphology to those of *S. beyi, Enhydriodon* sp. (Werdelin, Lewis & Haile-Selassie, 2014), and *A. capensis* (Figs. 6H–6K). However, it differs from the large *Enhydriodon* sp., in having a relatively smaller head and thinner neck, and a bigger distal projection of the astragalar tubercle. It is not known if that projection is also present in *S. beyi* because the preserved one lacks the astragalus tubercle (Peigné et al., 2008). While SAM-PQL-72172 is very similar to the astragalus of *A. capensis*, it also displays some differences, such as a shallower and mediolaterally wider trochlea, and a more medioproximal projected astragalar tubercle, including a more robust and deeper groove for the tendons of the plantar flexor muscles (Figs. 6H–6K). The relatively larger trochlea of *S. hendeyi* implies a relatively lateromedially wider distal tibia epiphysis, to that of living *A. capensis*. Based on the new astragalus (SAM-PQL-72172) (Figs. 3BB–3GG; Table 3), and its similarities with the astragal of *A. capensis* (Fig. 6), the astragalus SAM-PQL-50117 was correctly re-interpreted by Pickford (2005) as not belonging to an otter. The fragmentary radii (SAM-PQL-50001A and B) (Fig. 7) assigned by Hendey (1978b) to the bunodont otter from LBW, does not belong to *S. hendeyi*. A re-examination showed that SAM-PQL-50001A and B have a sharper and more distally projected styloid process, and a deeper, and enlarger groove for the tendon of the M. *abductor digiti I longus* to that of the living otter *A. capensis* (SAM-ZM-41474) (Figs. 7A–7J). Medially, a relatively large proximodistal crest-like projection is present, contrary to the round and reduced one present in SAM-ZM-41474. In distal view, both distal epiphyses are craniocaudally wide. However, SAM-PQL-50001A and B are distinguished to SAM-ZM-41474 in the smaller lateral groove for the tendon of the common digital extensor. All these traits suggest SAM-PQL-50001A and B cannot be assigned to *S. hendeyi* or other known mustelid from LWB on the basis of its morphological differences and measurements (Hendey, 1978b; Tables 4 and 5). Instead, SAM-PQL-50001A and B are much closer in morphology and size to the radius SAM-PQL-22061 of the giant sized viverrid cf. *Viverra leakeyi* Petter, 1963 from LBW (Figs. 7K–7O; Table 4).

Living lutrinae have a large shape diversity, which may be related to the large evolutionary and ecological variation of this group (Botton-Divet et al., 2016). While all species are semi-aquatic, the amount of time they spend in water differs with their type of habitat, swim, and food (Kruuk, 2006; Botton-Divet et al., 2016). The fragmentary nature of the known postcranial skeleton of *S. hendeyi* makes it difficult to suggest paleobiological interpretations about its locomotion or lifestyle. Previous interpretations were based entirely on its femur. The first one was made by Lewis (2008), who, based on the complete femur SAM-PQL-41523, inferred that this taxon could be a locomotor generalist in comparison with some other living and extinct Mio-Pliocene otters, but she did not reject the possibility of being semiaquatic, like extant river otters, or occasionally aquatic, like many non-lutrine mustelids. In the same year, Peigné et al. (2008) analyzed a relatively complete skeleton of *S*. *beyi* including the femur. He found that the femur of *S. beyi* was slenderer than that of *S. hendeyi*, and suggested that the Chadian specie was a terrestrial predator with poorly developed aquatic adaptations. The expanded sample of postcranial material of *S. hendeyi*, shows that this extinct otter shares more traits with *A. capensis* —for example, rostrocaudal compression of the distal epiphysis of the humerus, a more robust diaphysis of the femur, which suggests a relatively reduced total length (higher FRI), and a similar astragalus—than with *S. beyi*, indicating that *S. hendeyi* could be interpreted as a relatively more aquatic taxon than the former. Moreover, discussion of the paleoecology of extinct carnivores must take into account that all the categories described in the life history of the living ones such as cursorial, semifossorial, semiaquatic, arboreal, or terrestrial-generalist showing a complex continuum of behaviors none of them mutually exclusives (see Samuels, Meachen & Sakai, 2013; Fabre et al., 2015). Both semiaquatic and semifossorial carnivorans, have several shared anatomical traits, such as the rostrocaudal compression of the distal epiphysis of the humerus, relatively long olecranon process of the ulna and a medial projection of the diaphysis of the ulna for the attachment of the M. *pronator quadratus*, and an enlarged femoral epicondyles (Samuels, Meachen & Sakai, 2013; Fabre et al., 2015; Botton-Divet et al., 2016; Kilbourne & Hutchinson, 2019; Parsi-Pour & Killbourne, 2020; A. Valenciano, 2020, personal observations). We can preliminarily infer the locomotion or lifestyles of these extinct bunodont otters, based on the overall morphology of their skeletons. *Enhydriodon dikikae* and *S. beyi* can be interpreted as a more generalized terrestrial mustelid (Lewis, 2008; Peigné et al., 2008; Geraads et al., 2011) than *S. hendeyi*. Although more information about other bones are needed to assess the locomotion of *S. hendeyi*, we hypothesize it may have had a relatively more semiaquatic locomotion closer to that of the living *A. capensis* or *Am. cinereus*, but without excluding some digging capability. Similarly, *S. hendeyi* and *S. beyi* seems to have a lower association with water bodies than the North American *En. terraenovae* and *Sa. piscinarium* whose bones point to a more aquatic locomotion, similar to the living *Lutra* and *Pteronura* respectively (Bjork, 1970, Lambert, 1997).

Subfamily Guloninae Gray, 1825

Genus *Plesiogulo* Zdansky, 1924

**Type species:** *Plesiogulo brachygnathus* (Schlosser, 1903) by original designation.

**Other included species:** *Plesiogulo marshalli* (Martin, 1928); *Plesiogulo monspessulanus* Viret, 1939; *Plesiogulo crassa* Teilhard de Chardin, 1945; *Plesiogulo praecocidens* Kurtén, 1970; *Plesiogulo lindsayi* Harrison, 1981; *Plesiogulo botori* Haile-Selassie, Hlusko & Howell, 2004.

*Plesiogulo aff. monpessulanus* Viret, 1939

1978b *Plesiogulo monpessulanus*: Hendey, p. 330, figs.1, 2, 3, 4, 5, 6.

2016 *Plesiogulo monpessulanus*: Hartstone-Rose et al., p. 3, fig. 1.

**Locality:** Langebaanweg, early Pliocene, LQSM and MPPM.

**New material from Langebaanweg:** SAM-PQL-40117, edentulous left maxillary of an adult specimen with P2-4 and M1 alveoli; SAM-PQL-47086, edentulous left maxillary of a juvenile specimen including a fragmented DP4 and alveoli for P3-M1 and DP3; SAM-PQL-6246, left distal part of a humerus; SAM-PQL-L3440, right distal part of a radius; C. SAM-PQL-6414, right proximal fragment of an ulna.

**Description**

**Figure 8:**
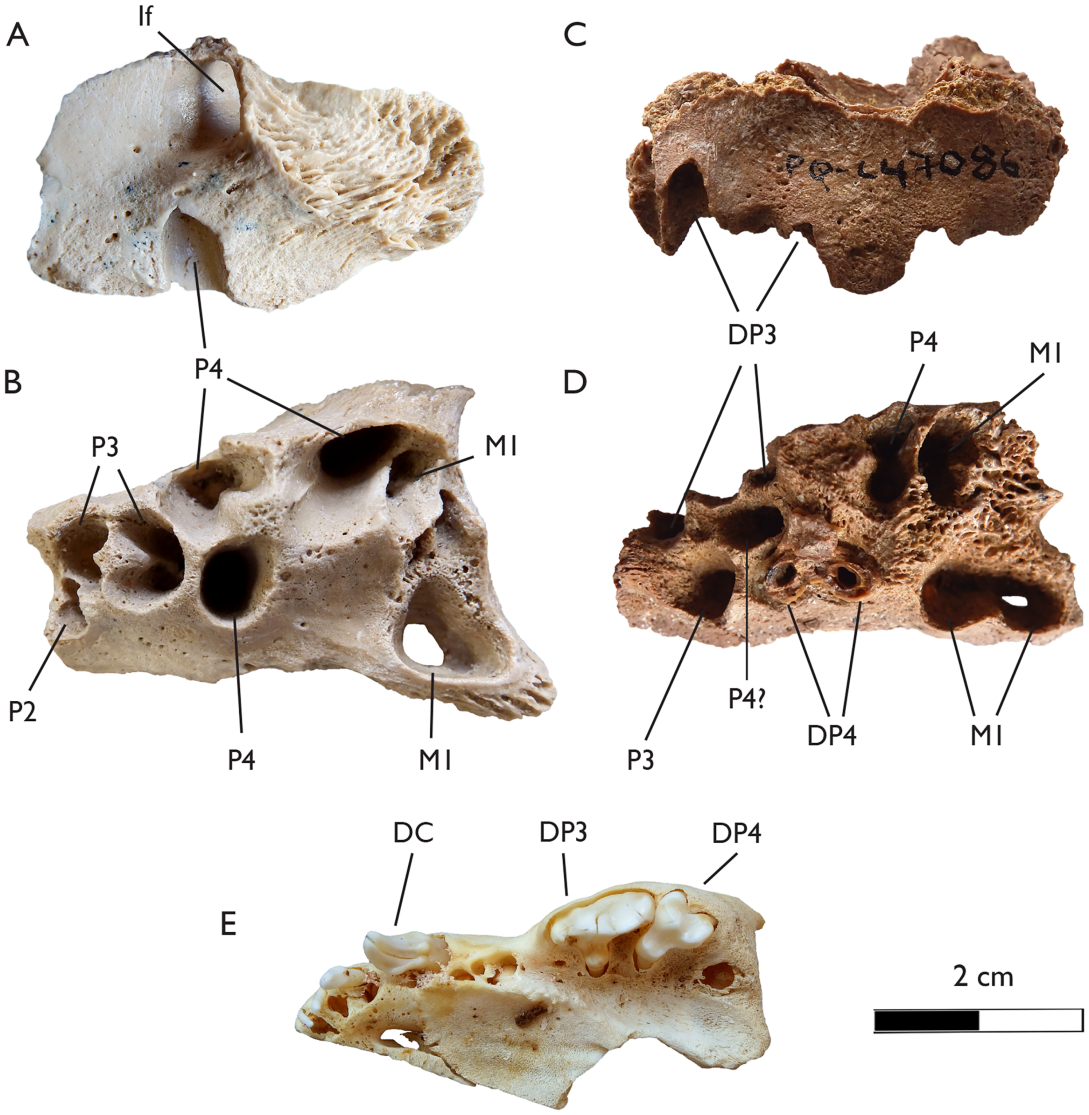
New maxillae of *Plesiogulo* aff. *monspessulanus* from Langebaanweg compared to a juvenile one of a living wolverine (*Gulo gulo*). (A and B) SAM-PQL-40117, edentulous left maxilla of an adult specimen of *Plesiogulo* aff. *monspessulanus* showing the alveolus. (A) Lateral, and (B) occlusal views. (C and D) SAM-PQL-47086, edentulous left maxilla of a juvenile specimen of *Plesiogulo* aff. *monspessulanus* showing the alveolus and a broken DP4. (C) lateral, and (D) occlusal views. (E) NRM-A587616, left maxilla of a juvenile specimen of *G. gulo*, occlusal view. The scale bar equals 2 cm. Abreviations: D, decidual dentition; If, infraorbital foramen, P, premolar, M, molar.

**SAM-PQL-40117:** edentulous left maxillary, comprising definitive alveoli (Figs. 8A and 8B). The bone surface is abraded. Laterally, there is a relatively large, oval infraorbital foramen (Fig. 8A). Below this foramen, the mesiobuccal root of the P4 is exposed as well as the cranial and basal part of the zygomatic arch in the caudal part of the maxilla (Fig. 8A). Compared with SAM-PQL-40042, where P2 possesses a double root, SAM-PQL-40117 only preserved the distal one (Fig. 8B). The alveolus for the P3 has two roots, and the P4 three. The M1 alveolus indicates that the M1 have an enlarged lingual area.

**SAM-PQL-47086:** edentulous left maxillary of a juvenile specimen, with an abraded surface. The buccal roots of the DP3 are present (Figs. 8C and 8D). It also preserves the lingual roots of the DP4. The lingual area is mesiodistally enlarged, with an incisures in the middle of the base of the lingual platform (Fig. 8D). It also has the distal root for the P3. It is not clear if the roots of the definitive P4 are present. Due to ontogenetic stage of SAM-PQL-47086, the definitive M1 was located deeper on the maxilla, which is broken in the occlusal plane, and allows the observation of the M1 alveolus. This alveolus differs from SAM-PQL-40117 (Fig. 8D). It is mesiodistally enlarged and shows a clear concavity in its middle point (Fig. 8D), reflecting the shape of the definitive M1, which is characteristic in *Plesiogulo*.

**Figure 9:**
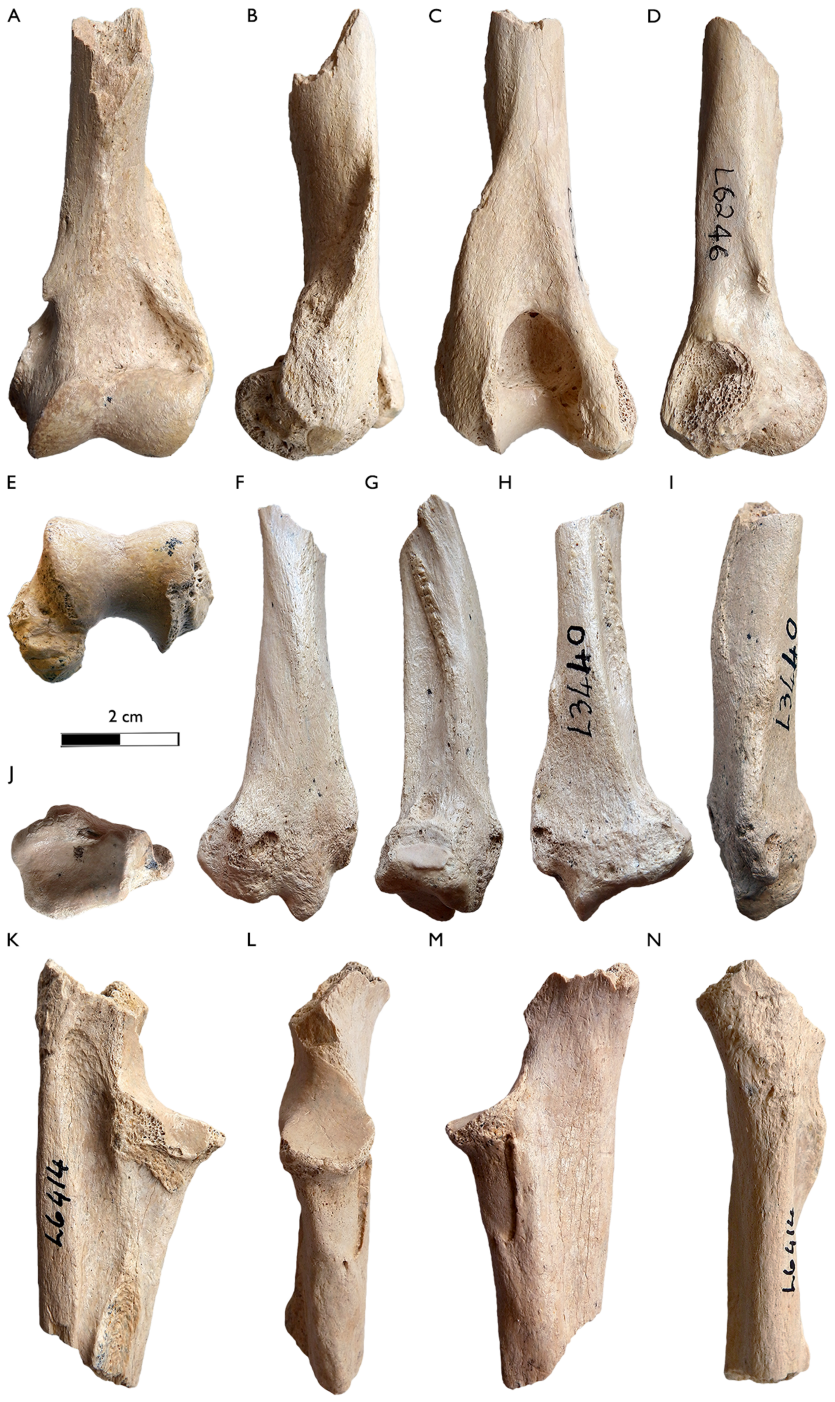
New postcranial remains of *Plesiogulo* aff. *monspessulanus* from Langebaanweg. (A–E) SAM-PQL-6246, left distal part of the humerus. (A) Rostral, (B) lateral, (C) caudal, (D) medial, and (E) distal views. (F–J) SAM-PQL-L3440, right distal part of the radius. (F) Rostral, (G) lateral, (H) caudal, (I) medial, and (J) distal views. (K–N) SAM-PQL-6414, right proximal fragment of the ulna. (K) Lateral, (L) rostral, (M) medial, and (N) caudal views. The scale bar equals 2 cm.

**SAM-PQL-6246:** fragmentary left humerus including the both distal part of the diaphysis and the distal epiphysis (Figs. 9A–9E) showing evidence of abrasion. There are longitudinal cracks along the main axis of the bone. It is smaller than the previously known humerus of *Plesiogulo* (SAM-PQL-40042). The cortical bone of the diaphysis is thick. It has a well-developed lateral epicondylar crest. The distal epiphysis is rectangular in distal view. The olecranon fossa is deep and proximodistally high. Medially, the supracondyloid process, is broken, so it only preserves half of the entepicondylar foramen. The medial epicondyle is abraded, but distally it is projected in caudal direction (Figs. 9D and 9E).

**SAM-PQL-L3440:** fragmentary, abraded right distal part of a radius, including the distal epiphysis (Figs. 9F–9J). The diaphysis is craniocaudally curved similar to the radius of *Plesiogulo* (SAM-PQL-40042). In cranial view, a crest is present exceeding half the diaphysis (Fig. 9F). It can be interpreted as a scar for the most distal attachment of the M. *supinator*. Laterally on the radius, an edge with a rough surface from the proximal-most part to the lateral border is present, occupying half of the preserved diaphysis (Fig. 9G). It is interpreted as the most distal part of the interosseous border. The ulnar notch is oval and craniocaudally elongated. The knob shaped medial styloid process is the insertion for the M. *brachioradialis* (Fig. 9I). The styloid process is pointed. In cranial view, there are three grooves on the cranial border of the distal epiphysis (Fig. 9H). The medial one is for the tendon of the M. *abductor digiti I*. A lateromedially enlarged one in the middle of the epiphysis, over the cranial border. It is relatively deeper and represent the groove for the tendon of the M. *extensor carpi radialis*. A smaller lateral groove exists for the tendon of the M. *extensor digitorum comunis*. The distal epiphysis is craniocaudally wide (Fig. 9J).

**SAM-PQL-6414:** fragmentary right proximal epiphysis of an ulna (Figs. 9K–9N). It is abraded and has longitudinal cracks along the main axis of the bone similar to the humerus (SAM-PQL-6246). The olecranon process is missing and the bone is broken proximally at the trochlear notch area, and distally at the most proximal part of the interosseous border. The overall shape and size of the preserved ulna is similar to the one of *Plesiogulo* (SAM-PQL-40042), especially the medial coronoid process and the insertion of the M. *brachialis*, located below this process, which is proximodistally enlarged (Figs. 9L and 9M). There is a very noticeable roughness for the interosseous border (Fig. 9K) in the lateral side of the bone.

**Discussion**

*Plesiogulo* is a large to very large mustelid known from late Miocene to early Pliocene localities in Eurasia, Africa and North America (Schlosser, 1903; Zdansky, 1924; Martin, 1928; Viret, 1939; Teilhard de Chardin, 1945; Petter, 1963; Kurtén, 1970; Hendey, 1978b; Harrison, 1981; Koufos, 1982; Morales, 1984; Rook, Ficcarelli & Torre, 1991; Alcalá, Montoya & Morales, 1994; Sotnikova, 1995; Barry, 1999; Haile-Selassie, Hlusko & Howell, 2004; Morales, Pickford & Soria, 2005; Koufos, 2006; Montoya, Morales & Abella, 2011; Morales, Pickford & Valenciano, 2016; Jiangzuo, Yu & Flynn, 2019; Grohé, in press). The systematic position of *Plesiogulo* within mustelidae and the living wolverines (*Gulo*) is complicated by several shared common craniodental traits and the long divergence time among these genera. Some researchers supported an ancestor-descendant relationship between Plesiogulo and Gulo (Viret, 1939; Kurtén, 1970; Kurtén & Anderson, 1980), whereas others considered that Plesiogulo is included in an extinct lineage without living descendants (Zdansky, 1924; Hendey, 1978b; Harrison, 1981; Xiaofeng & Haipo, 1987; Alcalá, Montoya & Morales, 1994; Sotnikova, 1995; Montoya, Morales & Abella, 2011; Samuels, Bredehoeft & Wallace, 2018). Valenciano et al. (2017b, 2020) support *Plesiogulo* and the early Miocene *Iberictis* forming a sister group of *Gulo* based on morphological traits and cladistic analysis; considering these three taxa as member of the tribe Gulonini. Molecular analyses agree with the existence of a “*Gulo* lineage” in which there is a close relationship between *Gulo*, *Martes* and *Pekania* among other taxa, with a divergence time for *Gulo* around 7.6–5.5 Ma (Koepfli et al., 2008; Li et al., 2014; Malyarchuk, Derenko & Denisova, 2015; Law, Slater & Mehta, 2018). It does not exclude *a priori* that extinct taxa like *Plesiogulo* may belong to this clade. Nevertheless, Samuels, Bredehoeft & Wallace (2018) reinforced a sister group relationship between *Gulo* and *Pekania* through a new species of *Gulo* from the early Pliocene (4.9–4.5 Ma) of Tennessee (USA), considering *Plesiogulo* to be convergent with *Gulo*. Thus, morphologically an Early-Late Miocene clade of mustelids comprising *Plesiogulo* is well documented (Valenciano et al., 2020), and the systematics position of *Gulo* is debatable, depending of the divergence time of *Gulo*-*Martes*-*Pekania* and the absence of a direct systematic relationship between *Gulo sudorus* Samuels, Bredehoeft & Wallace, 2018 and *Plesiogulo*.

There are three species of *Plesiogulo* of very large size: *P. monspessulanus* in Eurasia and South Africa (Viret, 1939; Teilhard de Chardin, 1945; Hendey, 1978b; Morales, 1984; Alcalá, Montoya & Morales, 1994; Montoya, Morales & Abella, 2011), *P. lindsayi* in U.S.A. (Harrison, 1981) and *P. botori* in Kenya and Ethiopia (Haile-Selassie, Hlusko & Howell, 2004; Morales, Pickford & Soria, 2005; Morales, Pickford & Valenciano, 2016). Hendey (1978b) described remains of three individuals of *P. monspessulanus* from LBW, comprising a fragmented skull, three hemimandibles and abundant postcranial remains. The type locality of *P. monspessulanus* is Sables de Montpellier, France (MN14, early Pliocene) (Viret, 1939). The only known material is the holotype, consisting of a partial right mandible with p3-4 and m1 without metaconid. This taxon has been recorded in the Iberian Peninsula by a M1, and both a m1-2 from Las Casiones, Spain, late Miocene, MN13, 6.3 Ma (Alcalá, Montoya & Morales, 1994; Gibert et al., 2013), and from Venta del Moro, Spain, late Miocene, MN13, 6.23 Ma, through a fragmentary P4 and a mandible with p2-4 and m1 (Morales, 1984; Montoya, Morales & Abella, 2011; Gibert et al., 2013). It also occurred in the early Pliocene (=Astian) of Yushé, China, where a mandible of *Plesiogulo major* was described by Teilhard de Chardin (1945) and later synonymized with *P. monspessulanus* by Hendey (1978b).

**Figure 10:**
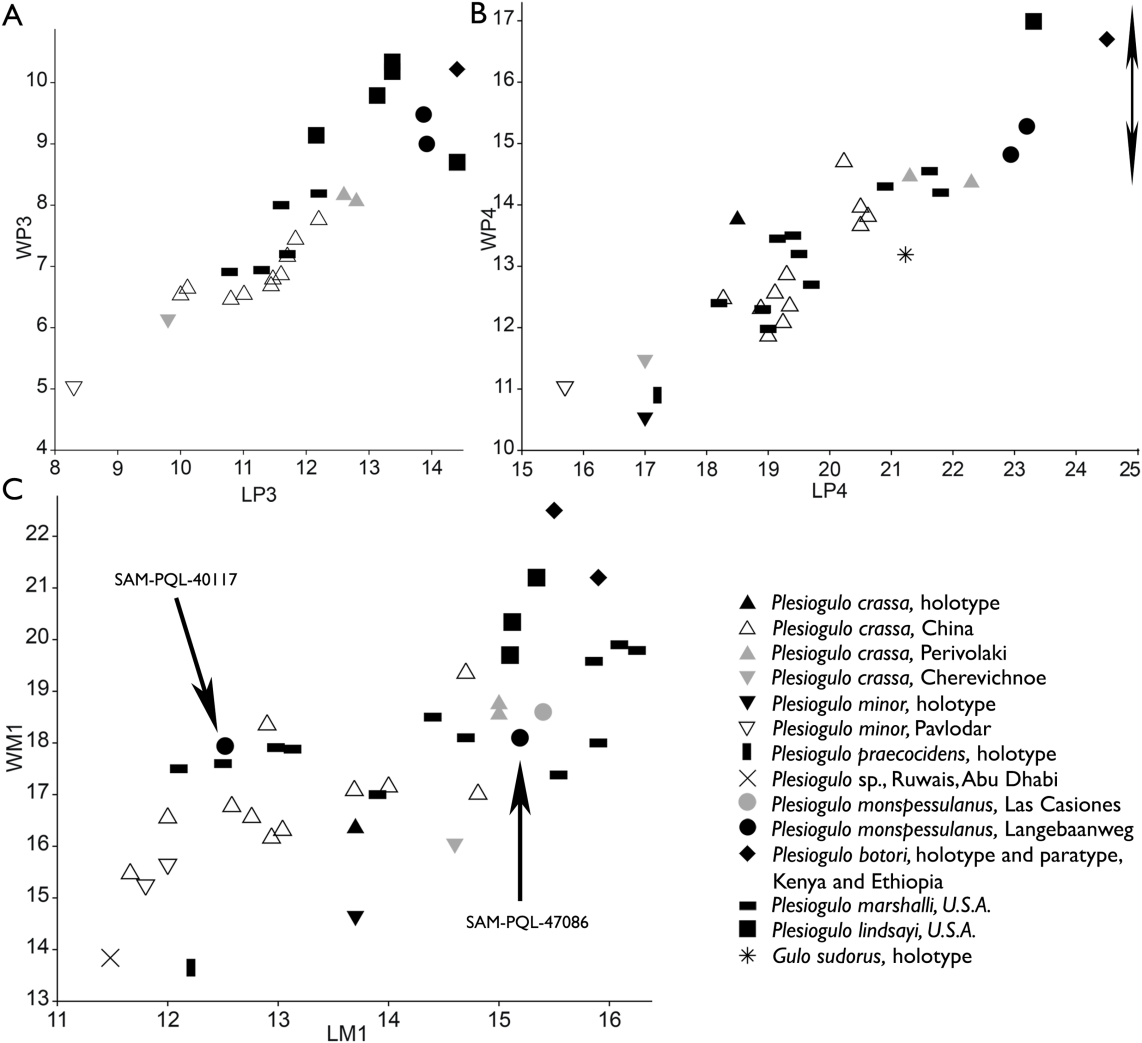
Measurements (mm) of the upper dentition of *Plesiogulo* spp., comprising the new material of Langebaanweg based on the alveoli, depicted by bivariate plots of maximum mesiodistal length (L) vs. maximum buccolingual width (W). (A) P3. (B) P4. The arrow indicated the hypothetical range of variation for the W of the P4 (*L* = 24 mm, see Morales, 1984) from Venta del Moro (Late Miocene, Spain). (C) M1. Estimation of the paratype of *P. botori* (M1 ADD-VP-1/10) based on picture provided in Haile-Selassie, Hlusko & Howell (2004). Sources: (Zdansky, 1924; Teilhard de Chardin, 1945; Kurtén, 1970; Hendey, 1978b; Harrison, 1981; Morales, 1984; Alcalá, Montoya & Morales, 1994; Sotnikova, 1995; Haile-Selassie, Hlusko & Howell, 2004; Koufos, 2006; Montoya, Morales & Abella, 2011; Samuels, Bredehoeft & Wallace, 2018; Grohé, in press; and this work).

The current novel material described represents the first new specimens of this taxon from the locality since the Hendey’s work in the 1970’s (Figs. 8 and 9). The new dental measurements of the P3 and P4 based on the alveoli and SAM-PQL-40042, point to this form falling among the largest specimens of the genus (Fig. 10; Tables 1 and 2). The morphology and proportions of the M1 of this mustelid from LBW is unknown. However, we can infer it based on the alveolus of SAM-PQL-47086. Although it belongs to a juvenile individual, it is noted that it was not erupted, and the preserved part of the alveolus in which the lingual platform was placed, represents a relatively accurate dimension for the maximum length of the M1. It also suggests that the lingual platform has an inflexion in the middle of the crown (Fig. 8D), being a distinctive trait for the genus. The inferred dimension of SAM-PQL-47086 are close to the M1 of *P. monspessulanus* from Las Casiones (Alcalá, Montoya & Morales, 1994) (Fig. 10; Table 2). SAM-PQL-47086 also preserved part of the upper deciduous dentition. The only previous deciduous dentition described in *Plesiogulo* is the lower one of the North American *P. marshalli* (Harrison, 1981), therefore it is impossible to make a direct comparison. Interestingly, the DP4 of SAM-PQL-47086 is similar to the definitive M1, which indicates the possession of an enlarged lingual platform, while the contrary pattern occurs in the living gulonini *G. gulo*, which DP4 and M1 are reduced (Figs. 8C–8E). These differences in the M1 was interpreted by Valenciano et al. (2020) as alternative strategies to crush bones; food processing in *Gulo* is more focused on the postcanine dentition and in the carnassials (P4-m1) and in *Plesiogulo* on the most distal dentition comprising the carnassials and the M1. In general terms, the proportions of the dentition of the South African *Plesiogulo* are close to the holotype of *P. monspessulanus* and the one from Las Casiones (Fig. 10; Tables 1 and 2). The m1 metaconid is absent in the holotype but it is present in the specimens from Las Casiones, Venta del Moro and Yushé (Viret, 1939; Teilhard de Chardin, 1945; Alcalá, Montoya & Morales, 1994; Montoya, Morales & Abella, 2011). The presence or absence of this trait in the one from LBW is not clear. The whole lingual part of the m1 of SAM-PQL-21570 is missing, and in SAM-PQL-28394 is quite worn. The worn area where the metaconid may be in the tooth, is widen at its base, suggesting it was really developed. This feature is variable in this genus and is not very useful for taxonomic analysis. However, the loss of the m1 metaconid in several caniform carnivorans such as canids, temnocyonine amphicyonids and mustelids has been interpreted as a derived trait (Van Valkenburgh, 1991; Hunt, 2011; Valenciano et al., 2016, 2017b, 2019), and in this case the presence of the metaconid in the oldest specimens from Las Casiones, Venta del Moro and the early Pliocene of Yushe indicate a more primitive stage for this trait. The classical material of *Plesiogulo* from LBW was found in LQSM (Hendey, 1978b; Werdelin, 2006), with the exception of SAM-PQL-40042, which according to Hendey (1978b) could come either from LQSM or from the lowermost levels of bed 3aS from MPPM. The new material confirms the presence of *Plesiogulo* in both LQSM and MPPM at LBW (Table 6).

**Figure 11:**
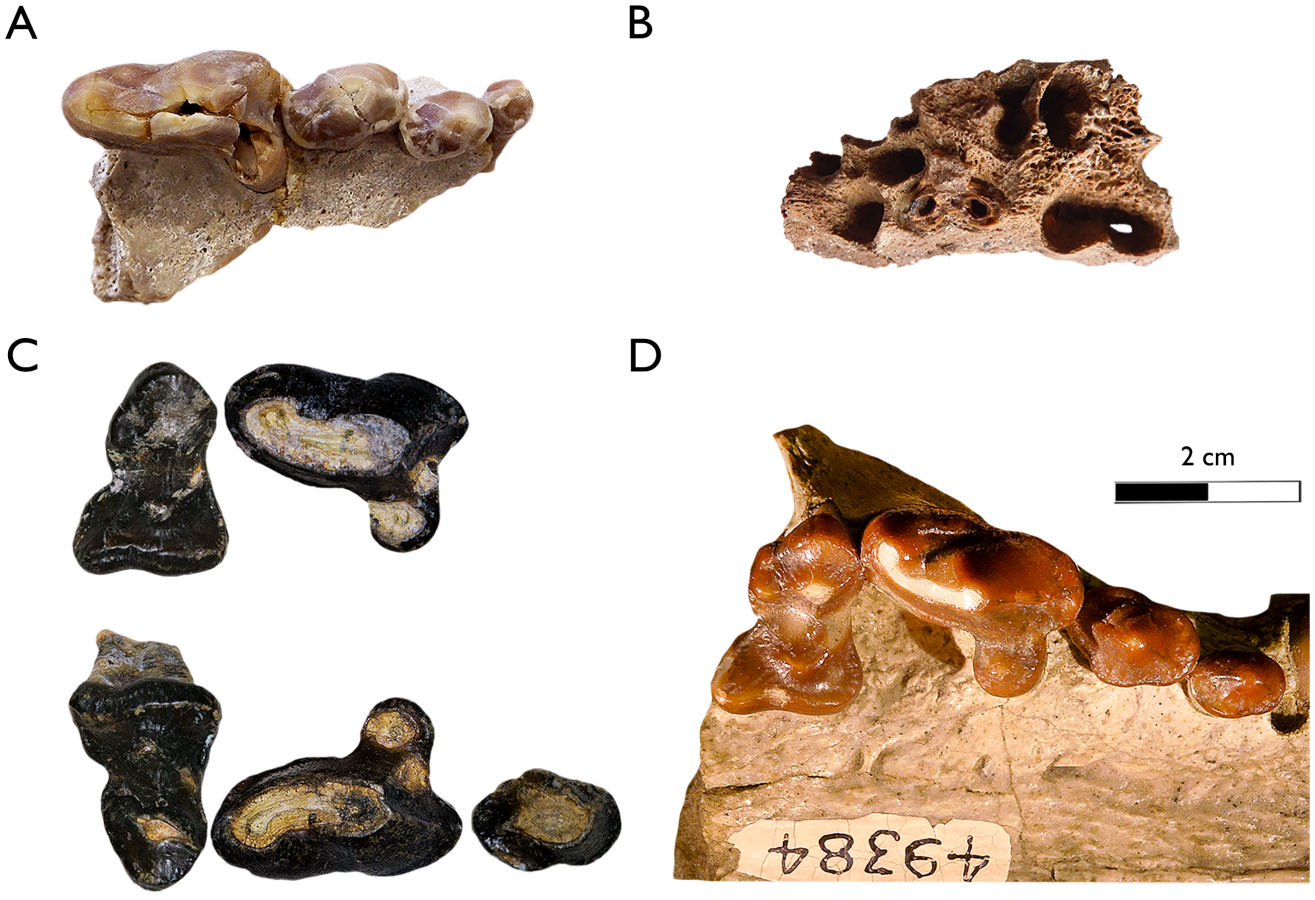
Main comparative material of very large-sized Plesiogulo spp in occlusal view. (A and B) *Plesiogulo* aff. *monspessulanus* from Langebaanweg. (A) SAM-PQL-40042, right fragmentary maxillary including P1-4. (B) SAM-PQL-47086, edentulous left maxilla of a juvenile specimen of *Plesiogulo* aff. *monspessulanus* from Langebaanweg showing the internal structure of the M1 alveolus. (C) KNM-NK 41420, holotype of *Plesiogulo botori*, associated partial upper dentition including left P3–M1, right P4–M1 from Narok, Lemudong’o, Kenya (c.a., 6-5.54 Ma). (D) F:AM 49384, partial view of the maxillary of the holotype of *Plesiogulo lindsayi* from Wikieup area, Arizona, U.S.A. (late Hemphillian Land Mammal Age). Scale bar equals 2 cm.

An additional very large species of *Plesiogulo* is present in Eastern Africa in deposits dated to 5.5–6.0 Ma (Haile-Selassie, Hlusko & Howell, 2004; Haile-Selassie et al., 2004). *Plesiogulo botori* occurs in the localities of Narok (type locality), Lemudong’o Fm., Kenya and in Adu Dora, Middle Awash, Afar Depression, Ethiopia (Haile-Selassie, Hlusko & Howell, 2004). It represents the largest species of the genus (Figs. 10 and 11). However, only the upper dentition is known. Additionally, more material of *Plesiogulo* was described in contemporaneous sediments of Kenya in the Lukeino Fm., aged from the late Miocene, 6.1–5.7 Ma (Morales, Pickford & Soria, 2005). They assigned to *P. praecocidens* a right complete P4 from the locality of Cheboit and a fragmented M1 from the locality of Kapcheberek (Morales, Pickford & Soria, 2005). Recently, after a revaluation of these teeth, Morales, Pickford & Valenciano (2016) excluded the P4 for the genus and re-assigned the fragmentary M1 to *P. botori*. The P3 and P4 of the maxilla of SAM-PQL-40042 are slender and smaller to those of *P. botori* (Fig. 11C). The P4 protocone is partially broken in the specimen SAM-PQL-40042, however the overall morphology and its proportions can be inferred and it is distinguishable to those of *P. botori* (Fig. 11). Based on the above, the taxonomy of the large *Plesiogulo* from LBW is complex. The largest species from Eurasia (*P. monspessulanus*) and Africa (*P. botori*) are represented by incomplete holotypes; consisting of lower or upper dentition respectively. When Hendey described the material from LBW in 1978, he assigned it to *P. monspessulanus* (Hendey, 1978b) as only the Euroasiatic species was known. Since then, new large *Plesiogulo* described from the late Miocene of the Iberian Peninsula have been assigned to *P. monspessulanus*, adding more variability to the taxon (Alcalá, Montoya & Morales, 1994; Montoya, Morales & Abella, 2011), although with certain doubt for the remains from Venta del Moro (Montoya, Morales & Abella, 2011). These findings are however still insufficient to confidently assign the LBW material to *P. monspessulanus*. In the absence of complete diagnostic dentition such as M1 and a better-preserved P4 and m1, to make a direct comparison with the holotypes of *P. botori* and *P. monspessulanus*, and based on the similarities with the known lower dentition of the later, we refer the LBW sample to *Plesiogulo* aff. *monsspesulanus*. Thus, two very large species of *Plesiogulo* were present in Africa during the Mio/Pliocene, *P. botori* in the Late Miocene of Eastern Africa spanning 6.1-5.5 Ma (Haile-Selassie, Hlusko & Howell, 2004; Haile-Selassie et al., 2004; Morales, Pickford & Soria, 2005; Morales, Pickford & Valenciano, 2016) and *Plesiogulo* aff. *monspessulanus* in the slightly younger deposits of Langebaanweg at the beginning of the Pliocene in Southern Africa (5.2 Ma).

The postcrania of *Plesiogulo* spp., are poorly known with the exception of the fragmentary forelimbs of *P. marshalli* and *P. lindsayi* described by Harrison (1981), and the abundant but fragmentary skeleton of *P*. aff. *monspessulanus* from LBW (Hendey, 1978b) (Table 5). The new forelimb remains complement the previous one of SAM-PQL-40042. These fragmentary humerus, ulna and radius, have a relatively equivalent size and preservation, which suggest they may belong to the same individual (Fig. 9; Table 5). They represent a smaller individual than SAM-PQL-40042, which also possesses the largest dentition of *Plesiogulo* from LBW. Based on the preservation and that they are from MPPM, they do not belong to the individuals SAM-PQL-28394 or SAM-PQL-21570, which came from LQSM. The overall morphology of the forelimb of *P*. aff. *monspessulanus* is robust. The humeri SAM-PQL-6246 and SAM-PQL-40042 are larger than the one of *P. marshalli* (Table 5), having a relatively better developed lateral epicondylar crest and a more enlarged medial epicondyle to those of the North American species. However, these differences can be interpreted as allometric differences because of the larger size of the South African taxon, instead of biomechanical implications. The ulnae SAM-PQL-6414 and SAM-PQL-40042 are quite similar to that of *P. marshalli* and *P. lindsayi*, but larger (Table 5). We calculated the olecranon length index (OLI) and ulnar robustness (URI) for the ulnae of *P*. aff *monspessulans* SAM-PQL-40042, *P. marshalli* and *G. gulo* following Samuels, Meachen & Sakai (2013) (Table 5). The OLI indicates the relative mechanical advantage of the M. *triceps brachii* used in the elbow extension (Samuels, Meachen & Sakai, 2013). The value of SAM-PQL-40042 is higher than *P. marshallli*, and equivalent to the scansorial Chinese ferret-badger *Melogale moschata* (Gray, 1831) and close to the semifossorial honey badger *Mellivora capensis* and the generalist *Melursus ursinus* (Shaw, 1791). The URI indicates the degree of robustness of the ulna and its ability to resist bending and shearing stresses and relative area available for the origin and insertion of forearm and manus flexors, pronators and supinators (Samuels, Meachen & Sakai, 2013). *Plesiogulo* aff. *monspessulanus* from LBW have a very robust ulna with the highest value for URI, only comparable with the saber-tooth felid *Smilodon fatalis* (Leidy, 1868), which was classified as terrestrial by Samuels, Meachen & Sakai (2013). Based on these indices *P*. aff *monspessulans* possesses a very robust ulna with a regular olecranon development. Hendey (1978b) described the skeleton of *P*. aff. *monspessulanus* from LBW as similar to *G. gulo*, but with the limb bones, tarsals and metapodials less elongated and more stoutly proportioned, which reflect the stoutness of this species and its heavier musculature. The curvature of the ulna SAM-PQL-40042 and the radii SAM-PQL-40042 and SAM-PQL-L3440 point toward this trend. The possession of a very robust forelimb in *P*. aff *monspessulans* could suggest that this species was an ambush predator, in a similar mode to that of the North American *Sm. fatalis* (Meachen-Samuels & Van Valkenburgh, 2010, Lewis, 2018), which would surprise and subdue their preys rather than chase them down.

## General Discussion and Conclusions

The Miocene–Pliocene boundary (5.33 Ma), represents a time of major turnover in carnivoran faunas in Eurasia and Africa, and several groups of carnivorans as percrocutids, and amphicyonids have their last records in Africa at that time (Werdelin & Turner, 1996, Van der Made, Morales & Montoya, 2006; Werdelin & Peigné, 2010; Gradstein et al., 2012; Werdelin & Lewis, 2017). This turnover is slightly delayed in Africa, with the persistence of characteristic Miocene taxa into the earliest Pliocene (Werdelin & Turner, 1996; Werdelin & Peigné, 2010; Werdelin & Lewis, 2017). Among them, the most notable late Miocene genera of Euroasiatic origin are the ursid *Indarctos*, the hemicyonid *Agriotherium* (considered an ursid by other authors), the mustelids *Plesiogulo, Sivaonyx* and *Enhydriodon*, the hyaenids, *Adcrocuta*, *Chasmaporthetes*, and *Hyaenictis*, the saber-tooth felids *Amphimachairodus* and *Metailurus*, and the canids *Vulpes* and *Eucyon* (Morales, Pickford & Soria, 2005; De Bonis et al., 2007; Werdelin & Peigné, 2010). The carnivoran sample of LBW is rich and diverse, including more than 19 different taxa of mustelids, canids, hemicyonids, phocids, hyaenids, felids, viverrids and herpestids (Hendey, 1974, 1978a, 1978b, 1980, 1982; Werdelin, Turner & Solounias, 1994; Werdelin & Lewis, 2001; Morales, Pickford & Soria, 2005; Morales & Pickford, 2005; Werdelin, 2006; Werdelin & Sardella, 2007; Govender, 2015). Among them, it is notable that several large carnivorans from the end of the Miocene were immigrants from Eurasia, including *Agriotherium*, *Amphimachairodus*, *Metailurus, Plesiogulo, Sivaonyx, Eucyon*, and *Hyaenictis*. All except *Eucyon* and *Sivaonyx* went extinct through the Pliocene, where these forms were replaced by more derived hyenas (*Chasmaporthetes, Ikelohyaena, Pachycrocuta*), canids (*Nyctereutes*, *Canis*, *Lupulella*), and felids (*Dinofelis*, *Megantereon*, *Homotherium*) (Werdelin & Peigné, 2010; Werdelin & Lewis, 2017).

The mustelid guild from this locality comprises the very large *P.* aff. *monspessulanus*, and *S. hendeyi*, as well as the smaller honey badger *Mellivora benfieldi* Hendey (1978b). Both *Plesiogulo* and *Sivaonyx* from LBW, are typical member of the Euroasiatic carnivore guild and have been recognized outside Africa before LBW. *Plesiogulo monspessulanus* occurred in Western Europe (Alcalá, Montoya & Morales, 1994; Morales, 1984; Rook, Ficcarelli & Torre, 1991; Rook et al., 2011; Montoya, Morales & Abella, 2011), and *Sivaonyx* spp., were found in older sediments in Africa and Asia (Peigné et al., 2008; Pickford, 2007; Grohé et al., 2013). After the previous dispersal event, an array of new species of large bunodont otter (*Sivaonyx* and *Enhydriodon*) and one new species of *Plesiogulo* diversified in East and South Africa (Haile-Selassie, Hlusko & Howell, 2004; Haile-Selassie et al., 2004; Morales, Pickford & Soria, 2005; Howell & García, 2007; Haile-Selassie, 2008; Haile-Selassie & Howell, 2009; Morales, Pickford & Valenciano, 2016). *Sivaonyx* and *Enhydriodon* were much more successful than the large wolverine, which went extinct at the beginning of the Pliocene.

The co-occurrence of three mustelids in LBW can be explained by dietary resource/ecological niche partitioning, in which none of them seem overlap. It has been suggested that *Plesiogulo* was an inhabitant of open, and grassy plains (Kurtén, 1970; Harrison, 1981), with the role of an ambush predator, with durophagous abilities (Valenciano et al., 2020). Werdelin (2015) stated that we can only speculate about the habits and diet of extinct bunodont otters, because they differ from any living relatives, highlighting that they appear to have been somewhat more terrestrial than living otters, but they are always found in association with large bodies of water. Even though the preserved craniomandibular remains of *S. hendeyi* are not complete, following the ecomorphological analysis of extant small carnivorans of Friscia, Van Valkenburgh & Biknevicius (2007), which include the extant otters *Enhydra* and *Amblonyx*, which feed on mollusks and crustaceans, it can be inferred that *S. hendeyi* possesses a comparable dentition, including larger molar grinding areas, larger post-canine dentitions, and wider fourth premolars, typical of omnivores/hard-object feeders. Also, a diet based on relatively hard items, as in the living *Aonyx capensis* has been previously suggested for other African bunodont otters (*Sivaonyx* spp., and *Enhydriodon* spp.) hypothesizing that they fed on armored catfishes, mollusks, and other armored preys (Pickford, 2007; Peigné et al., 2008; Geraads et al., 2011; Werdelin, 2015; Werdelin & Lewis, 2017). *Sivaonyx hendeyi* could have had a similar role to that of the living *A. capensis*, having a locomotion relatively similar to it while less semiaquatic. Its bunodont and robust dentition suggests an even more durophagous diet to those of *A. capensis*; and *M. benfieldi* can be interpreted as a small-medium opportunistic carnivoran analogous to the living honey badger *M. capensis*.

In previous studies (Hendey, 1972, 1974, 1976, 1978a, 1978b, 1980, 1981a, 1982; Werdelin, 2006), significant differences between the carnivoran faunas of the MPPM and LQSM has been highlighted. In the case of Mustelidae, *P*. aff. *monspessulanus* was found in LQSM, and *S. hendeyi* and *M. benfieldi* were found in MPPM (Hendey, 1974, 1978b, 1982; Werdelin, 2006). These faunal differences have been interpreted as due to temporal differences and faunal replacement (Hendey, 1972, 1974, 1976, 1978a, 1978b, 1980, 1981a, 1982). The re-study of the new material indicates these three mustelids are present in both members (Table 6; A. Valenciano, 2020, personal observations), suggesting that the differences observed previously may be produced by sedimentation (estuarine/marine/fluvial deposition) or sampling biases, instead of temporal replacement of the carnivoran guild. This is supported by a same estimated age of ~5.15 ± 0.1 Ma for both LQSM and MPPM, and by sedimentological, petrographical and geochemical evidences (Middleton, 2000, 2003; Pether, Roberts & Ward, 2000; Roberts et al., 2011). Thus, It is essential for there to be a taxonomic review of the other families from LBW before interpreting these faunal differences, especially the least analyzed canids, felids, viverrids and herpestids.

## Supplemental Information

10.7717/peerj.9221/supp-1 Supplemental Information 1 Measurements used in Figure 10

## Abbreviations

AMNH: American Museum of Natural History, New York, USA
SPG: Bayerische Staatssammlung für Paläontologie und Geologie, Munich, Germany
AM: collection housed in the Frick Collection of the Division of Paleontology, AMNH, New York, USA
CPT: Fundación Conjunto Paleontológico de Teruel-Dinópolis, Museo Aragonés de Paleontología, Teruel, Spain
MNH: Field Museum of Natural History, Chicago, USA
FSL: Université Claude Bernard Lyon 1, Lyon, France
SAM: Iziko South African Museum, Cape Town, South Africa
NM: Nairobi National Museum, National Museums of Kenya, Nairobi, Kenya
GUV: Museu de Geologia de la Universitat de València, Burjassot, Spain
MNCN: Museo Nacional de Ciencias Naturales Madrid, Spain
NRM: Naturhistoriska rikmuseet, Stockholm, Sweden
PMU: Palaeontological Museum, University of Uppsala, Uppsala, Sweden
SAM-PQL: Quaternary Palaeontology (Langebaanweg), Iziko South African Museum, Cape Town, South Africa
SAM-ZM: Zoology Mammals, Iziko South African Museum, Cape Town, South Africa
UF: Vertebrate Paleontology Collection of the Florida Museum of Natural History (FLMNH)
USNM: Smithsonian National Museum of Natural History (The Smithsonian Museum Support Center, Division of Mammals MSC, Suitland, USA)

## Additional Information and Declarations

### Data Availability

The following information was supplied regarding data availability: The measurements are available in Tables 1–5. AMNH: American Museum of Natural History, New York, USA; F:AM: collection housed in the Frick Collection of the Division of Paleontology, AMNH, New York, USA; FCPT: Fundación Conjunto Paleontológico de Teruel-Dinópolis, Museo Aragonés de Paleontología, Teruel, Spain; FMNH: Field Museum of Natural History, Chicago, USA; ISAM: Iziko South African Museum, Cape Town, South Africa; MGUV: Museu de Geologia de la Universitat de València, Burjassot, Spain; MNCN: Museo Nacional de Ciencias Naturales Madrid, Spain; NRM: Naturhistoriska rikmuseet, Stockholm, Sweden; PMU: Palaeontological Museum, University of Uppsala, Uppsala, Sweden; SAM-PQL: Quaternary Palaeontology (Langebaanweg), Iziko South African Museum, Cape Town, South Africa; SAM-ZM: Zoology Mammals, Iziko South African Museum, Cape Town, South Africa; UF: Vertebrate Paleontology Collection of the Florida Museum of Natural History (FLMNH); USNM: Smithsonian National Museum of Natural History (The Smithsonian Museum Support Center, Division of Mammals MSC, Suitland, USA). • *Sivaonyx hendeyi*: SAM-PQL-52861, SAM-PQL-50000A, B, C, SAM-PQL-9138, SAM-PQL-69635, SAM-PQL-72229, SAM-PQL-50120, SAM-PQL-60416, SAM-PQL-21264, SAM-PQL-72172, SAM-PQL-41523. • *Plesiogulo* aff. *monspessulanus*: SAM-PQL-40117, SAM-PQL-47086, SAM-PQL-40042, SAM-PQL-21570, SAM-PQL-28394, SAM-PQL-6246, SAM-PQL-3440, SAM-PQL-6414. • cf. *Viverra Leakeyi*: SAM-PQL-50001. • *Aonyx capensis*: SAM-ZM-41474, SAM-ZM-41483. • *Mellivora capensis*: SAM-ZM-41483, SAM-ZM-41666. • *Gulo gulo*: MNCN-16748; USNM 275160, USNM 272316, USNM A06231, USNM 265649, USNM 242705, USNM 108654, USNM 096147; NRM-A825005, NRM-A845012, NRM-20055154, NRM-20115498, NRM-A815010, NRM-A587719, NRM-A885007, NRM-A795005, NRM-A825004; FMNH-151027, FMNH-129317. • *Enhydritherium terraenovae*: UF100000. • *Plesiogulo crassa*: PMU-M16, PMU-M17, PMU-M18, PMU-M3846, PMU-M3805, PMU-M3806, PMU-M3807, PMU-M15, PMU-M20, PMU-M8132, PMU-M8136, PMU-M8128, PMU-M8129, PMU-M21. • *Plesiogulo monspessulanus*: MGUV-VV16615, and FCPT-KS-2. • *Plesiogulo praecocidens*: PMU-M19. • *Plesiogulo lindsayi*: AMNH 49384, F:AM 49388, F:AM 49387, F:AM 49370, F:AM 108060. • *Plesiogulo marshalli*: F:AM 108052, F:AM 67650A, AMNH 105237, F:AM 23378, F:AM 49490, AMNH 38902, F:AM 49491, F:AM 49494, F:AM 49498, F:AM 49479, AMNH 105449, F:AM 49230, AMNH 99599.
