# Supplementary material for: New insights into the giant mustelids (Mammalia, Carnivora, Mustelidae) from Langebaanweg fossil site (West Coast Fossil Park, South Africa, early Pliocene)
Source: PeerJ. 2020 Jun 1;8:e9221. doi: 10.7717/peerj.9221 (PMC7271888; doi:10.7717/peerj.9221)
Supplement: Supplemental Information 1 [file peerj-08-9221-s001.docx]

| Taxa/ Specimen | | Locality | P3 | | P4 | | M1 | |
| --- | --- | --- | --- | --- | --- | --- | --- | --- |
|  |  |  | L | W | L | W | L | W |
| ***Plesiogulo* aff. *monspessulanus*** | |  |  |  |  |  |  |  |
| SAM-PQL-47086 | | Langebaanweg |  |  |  |  | (15.2) | (18.1) |
| SAM-PQL-40117 | |  | (13.9) | (9.5) | (22.9) | (14.8) | (12.5) | (17.9) |
| SAM-PQL-40042* | |  | 13.9 | 9.0 | 23.2 | 15.3 |  |  |
| ***Plesiogulo monspessulanus*** | |  |  |  |  |  |  |  |
| KS-2 | | Las Casiones |  |  |  |  | 15.4 | 18.6 |
| MNCN ZV 215 | | Venta del Moro |  |  | 24.0 |  |  |  |
| ***Plesiogulo botori*** | |  |  |  |  |  |  |  |
| KNM-NK-41420 (holotype) | | Narok, Lemudong’o Fm. | 14.4 | 10.2 | 24.5 | 16.7 | 15.9 | 21.2 |
| ADD-VP-1/10** | | Dora (Middle Awash) |  |  |  |  | 15.5 | 22.5 |
| BAR- 1893’00 | | Kapcheberek, Lukeino Fm. |  |  |  |  | 14.6 |  |
| ***Plesiogulo lindsayi*** | |  |  |  |  |  |  |  |
| AMNH 49384 left | | Wikieup | 13.4 | 10.3 |  |  |  |  |
| AMNH 49384 right | | Wikieup | 13.4 | 10.2 | 23.3 | 17.0 | 15.3 | 21.2 |
| F:AM 49385 | | Wikieup | 12.2 | 9.1 |  |  | 15.1 | 20.3 |
| F:AM 49386 | | Wikieup | 13.1 | 9.8 |  |  |  |  |
| F:AM 50690 | | Old Cabin | 14.5 | 8.7 |  |  | 15.1 | 19.7 |
| ***Plesiogulo marshalli*** | |  |  |  |  |  |  |  |
| KUVP 3465 | | Edson Quarry |  |  | 19.4 | 13.5 | 14.4 | 18.5 |
| F:AM 49490 | | Optima | 11.6 | 8 | 20.9 | 14.3 | 15.9 | 18 |
| F:AM 23379 | | Coffee Ranch | 11.7 | 7.2 | 19.5 | 13.2 | 14.7 | 18.1 |
| F:AM 105237 | |  |  |  |  |  | 16.1 | 19.9 |
| UOMNH F-3656 | | McKay reservoir |  |  | 19.7 | 12.7 | 13.9 | 17 |
| UF 19253 | | Bone Valley |  |  | 21.8 | 14.2 | 12.1 | 17.5 |
| UF 19295 | |  |  |  |  |  | 12.5 | 17.6 |
| F:AM 49230 | | San Juan Quarry |  |  | 18.9 | 12.3 | 13.0 | 17.9 |
| KUVP 12433 | | San Lost Quarry |  |  | 18.2 | 12.4 |  |  |
| F:AM 23378 | | Miami Quarry | 12.2 | 8.2 | 21.6 | 14.5 | 15.9 | 19.6 |
| F:AM 23387 | |  | 11.3 | 6.9 | 19 | 12.0 | 13.1 | 17.9 |
| F:AM 23386 | |  | 10.8 | 6.9 | 19.1 | 13.4 | 15.5 | 17.4 |
| F:AM 23378B | |  |  |  |  |  | 16.2 | 19.8 |
| ***Plesiogulo crassa*** | |  |  |  |  |  |  |  |
| Holotype |  | Yushe, Turolian, China (Teilhard de Chardin, 1945) |  |  | 18.5 | 13.8 | 13.7 | 16.4 |
| PMU M16 (Ex1) left | | Loc.30 Baode Xian, Shansi, China | 10.0 | 6.6 | 19.2 | 12.1 | 12.9 | 16.2 |
| PMU M16 right | |  | 10.1 | 6.7 | 19.3 | 12.4 | 13.0 | 16.4 |
| PMU M3846 (Ex10) left | | Loc.111, China | 11.4 | 6.7 | 18.9 | 12.3 | 12.6 | 16.8 |
| PMU M3846 right | |  | 11.5 | 6.8 | 19.1 | 12.6 | 12.8 | 16.6 |
| PMU M3805 (Ex8) | | Loc. 108, China | 11.8 | 7.5 | 20.2 | 14.7 | 14.8 | 17.1 |
| PMU M20 | | Loc 30 (5), China |  |  | 20.6 | 13.8 | 13.7 | 17.1 |
| PMU M15 (Ex6) | | Loc. 108, China | 11.0 | 6.6 | 18.3 | 12.5 | 11.7 | 15.5 |
| Ex2 |  | Turolian, China (Zdansky, 1924) | 11.7 | 7.2 | 20.5 | 13.7 | 16.3 |  |
| Ex3 |  |  |  |  | 19.3 | 12.9 |  |  |
| Ex4 |  |  | 11.6 | 6.9 |  | 12.8 | 14.0 | 17.2 |
| Ex7 |  |  | 11.4 |  |  |  | 12.9 | 18.4 |
| Ex9 |  |  | 10.8 | 6.5 | 19.0 | 11.9 | 12.0 | 16.6 |
| Ex11 |  |  | 12.2 | 7.8 | 20.5 | 14.0 | 14.7 | 19.4 |
| PER-1239 left | | Perivolaki | 12.6 | 8.2 | 21.3 | 14.5 | 15.0 | 18.8 |
| PER-1239 right | |  | 12.8 | 8.1 | 22.3 | 14.4 | 15.0 | 18.6 |
| N 45/3271 | | Cherevichnoe | 9.8 | 6.1 | 17.0 | 11.4 | 14.6 | 16.0 |
| ***Plesiogulo minor*** | |  |  |  |  |  |  |  |
| Coll. Licent No. 553 | | K´ingyang, Kansu, China |  |  | 17.0 | 10.5 | 13.7 | 14.6 |
| ***Plesiogulo praecocidens*** | |  |  |  |  |  |  |  |
| PMU M20 (Ex5) | | Loc. 49, Paote, Shansi, China, |  |  | 17.2 | 10.9 | 12.2 | 13.6 |
| ***Plesiogulo* sp.** | |  |  |  |  |  |  |  |
| AUH 1762 |  | Ruwais, Abu Dhabi |  |  |  |  | (11.5) | 13.8 |

Supplemental table. Upper tooth measurements in mm of *Plesiogulo* from Langebaanweg (SAM-PQL), compared to other similar *Plesiogulo* species from figure 10. L= length, W= width. Parenthesis means measurements on alveoli or at the base of the broken crown. *= new measurement or re-measured after Hendey (1978a); *Pleiogulo botori* from Lukeino Fm. reasigned in Morales, Pickford & Valenciano 2016. Symbol: **= measured from the picture of Haile-Selassie, Hlusko & Howell, 2004a. Source (see Figure 10).
